# Pan-genome analysis reveals hidden diversity and selection signatures of auxin response factors (ARFs) associated with breeding in barley

**DOI:** 10.1007/s00122-026-05234-5

**Published:** 2026-04-20

**Authors:** Kenan Tan, Zhenru Guo, Thorsten Schnurbusch

**Affiliations:** 1 Leibniz Institute of Plant Genetics and Crop Plant Research (IPK), Corrensstraße 3, OT Gatersleben, 06466 Seeland, Germany; 2 Faculty of Natural Sciences III, Institute of Agricultural and Nutritional Sciences, Martin Luther University Halle-Wittenberg, 06120 Halle (Saale), Germany

## Abstract

Auxin response factors (ARFs) play a pivotal role in regulating plant growth and development; yet, their evolutionary dynamics and functional divergence remain poorly understood in barley (*Hordeum vulgare* L.) In this study, we conducted a comprehensive genome-wide analysis of the ARF gene family across a 76-accession barley pan-genome. By integrating gene presence/absence variation (PAV) and copy number variation (CNV), phylogeny, expression profiling, transposable element (TE)-mediated regulation, and selection signatures, we characterized 1,911 ARF-coding genes and their structural, transcriptional, and functional variation at the population level. Phylogenetic analysis revealed lineage-specific expansion and dynamic duplications, particularly within the HvARF13 clade. Co-expression networks and tissue-resolved transcriptomes showed that many *HvARFs* are preferentially expressed in inflorescence and meristematic tissues. Selective sweep and haplotype analyses identified *HvARF3* as a candidate gene under selection during European barley breeding. A favorable haplotype of *HvARF3*, enriched in European cultivars, was significantly associated with increased grain size and weight, demonstrating the utility of pan-genome-enabled frameworks for accelerating gene-trait association and candidate gene discovery. This study highlights the power of multi-omics integration in decoding gene family complexity and provides valuable insights for functional genomics and trait improvement in cereal crops.

**Supplementary Information:**

The online version contains supplementary material available at 10.1007/s00122-026-05234-5.

## Introduction

Auxin response factors (ARFs) are a family of plant-specific transcription factors that mediate auxin signaling by binding to auxin-responsive elements in gene promoters, thereby regulating a broad array of developmental and physiological processes. These include organogenesis, embryogenesis, apical dominance, root and shoot development, and reproductive differentiation (Cancé et al. 2022). Typically, ARF proteins harbor few functional domains: a conserved N-terminal B3 DNA-binding domain (DBD), a variable middle region that acts as either a transcriptional activator (glutamine-rich, Q-rich) or repressor (serine/threonine/proline-rich, STP-rich), and an optional C-terminal PB1 (Phox and Bem1) domain that facilitates homo- and hetero-dimerization with other ARFs or Aux/IAA proteins (Chandler 2016). This domain architecture is remarkable conserved from early land plants to higher angiosperms, underscoring the fundamental roles ARFs play across plant evolution (Li et al. 2023).

In numerous species—such as *Arabidopsis thaliana*, rice (*Oryza sativa* L.), maize (*Zea mays* L.), and wheat (*Triticum aestivum* L.)—*ARF* genes have been systematically identified and partially functionally characterized (23 *ARFs* in Arabidopsis; 25 *ARFs* in rice; 35 *ARFs* in maize; 52 *ARFs* in wheat) (Gidhi et al. 2021; Man et al. 2025; Okushima et al. 2005; Wang et al. 2007; Xing et al. 2011). These studies revealed the involvement of specific ARF members in regulating key agronomic traits such as grain size, inflorescence architecture, and stress responses. For example, *OsARF3, OsARF4, OsARF6, ZmARF24, ZmARF2, ZmARF17, and TaARF25* have been reported to regulate grain/kernel traits (Gao et al. 2023; Gu et al. 2023; Hu et al. 2018; Jia et al. 2021; Qiao et al. 2021; Wang et al. 2024). Notably, *OsARF3* is also involved in balancing the trade-off between abiotic and biotic stress responses. *OsARF12*, *OsARF17*, *OsARF25*, and *OsARF10* have been shown to affect stigma development (Guo et al. 2022; Zhao et al. 2022). A single amino acid substitution in *ZmARF28* can pleiotropically modify plant architecture, showing reduced plant height, increased leaf number and altered inflorescence identity (Prigge et al. 2025). These functional insights demonstrate the evolutionary versatility and their potential for agronomic improvement of *ARF* genes across cereals. Despite its agronomic and historical significance, as one of the first domesticated crops (Lister et al. 2018). While ARF genes have been functionally characterized in several cereals, their study in barley (*Hordeum vulgare* L.) remains limited. Previous analyses of the barley ARF family were restricted to a single reference genome based on early genome assemblies. Consequently, an updated and comprehensive assessment based on recent pan-genomic resources is essential. Given the known roles of ARF genes in other species, elucidating the diversity and function of the ARF protein family in barley could reveal previously unknown regulators of important developmental processes and contribute directly to crop improvement efforts.

Recent advances in high-throughput sequencing and the development of pan-genome and pan-transcriptome resources have transformed plant genomics by enabling species-wide resolution of genetic diversity (Marks et al. 2021). Traditional single reference-based analyses are insufficient to capture the full extent of gene family variation, as they often miss presence/absence variants (PAVs), copy number variations (CNVs), or population-specific alleles. Pan-genomic resources—such as those recently generated for barley—offer a more comprehensive view of structural and functional diversity at both the gene and genome levels (Jayakodi et al. 2024; Jayakodi et al. 2020). In parallel, transcriptome datasets across tissues, genotypes, and even at single-cell resolution now make it possible to explore gene expression divergence, regulation, and potential adaptation at an unprecedented scale.

In this study, we performed a comprehensive, population-scale analysis of the ARF protein family in barley using integrated multi-omics data. We systematically identified and annotated genes and proteins of the ARF family across the 76 barley pan-genomes, revealing approximately 30% more members than in previous studies (Tombuloglu 2019). By constructing high-resolution phylogenies and characterizing structural variation, expression patterns, *cis*-regulatory elements, and selective signals, we explored the evolutionary and functional dynamics of ARFs in barley. Our findings revealed significant differences in natural selection pressures and genomic diversity across distinct barley populations. Notably, we identified a superior allele of *HvARF3* that appears to have undergone selection in European cultivars, with significant association to increased grain size and weight. These findings demonstrate how integrative pan-genome and transcriptome analyses can illuminate both conserved gene family evolution and novel, population-specific adaptations with agronomic relevance. Our results provide a comprehensive panoramic view of the ARF protein family and establish a foundation for future studies on auxin signaling, inflorescence development, and molecular breeding in barley.

## Methods

### Identification of ARF genes in 76 barley pan-genome accessions

Genomic and annotated protein/coding sequences for 76 barley pan-genome accessions (including 23 wild accessions, 36 landraces, and 17 cultivars) were retrieved from a previously published study (Jayakodi et al. 2024). To comprehensive identify members of the ARF protein family, all candidate proteins containing ARF-related domains—including PTHR31384 from PANTHER; PF02362, PF06507, and PF02309 from Pfam, along with the B3 (PS50863) and PB1 (PS51745) domains—were identified using *HMMER* v3.1b2 (Finn et al. 2011) with an E-value threshold of < 1e−5. Domain annotations were verified by comparing against known ARF proteins from rice and *Arabidopsis* using *BLAST+* v2.13.0 (Camacho et al. 2009).Gene names (based on cv. Barke) were assigned according to phylogenetic relationship with rice *ARF* orthologs, which were identified through reciprocal BLASTP searches and confirmed by phylogenetic analysis. To confirm presence/absence variations (PAVs) and copy number variations (CNVs), we extracted 5 kb flanking sequences of loci where annotated ARF genes were missing using *Bedtools* v2.29.2 (Quinlan and Hall 2010). These regions were aligned to the Morex reference genome using *Minimap2* v2.16 (Li 2018) with default parameter. Large deletions were visualized using *MUMmer* v3.23 (Delcher et al. 2003) with default parameter, while smaller deletions were confirmed through BLAST alignments.

### Phylogenetic tree construction and *Ka*/*Ks*, Tajima’s D calculation

A total of 1,911 ARF protein sequences from the pan-genome were aligned using MAFFT v7.490 (Katoh and Standley 2013). Full length of transcript 1 of each ARF genes was used in the phylogenetic analysis. Phylogenetic relationships were inferred using *IQ-TREE* v2.2.2.6 (Nguyen et al. 2015) with the maximum likelihood method under -m MFP and other default parameters. For interspecies comparison, ARF protein sequences from barley (cv. Barke, BPGv2), rice (cv. Nipponbare, osa1 r7), and *Arabidopsis* (Col-0, TAIR10) were used to construct a combined phylogenetic tree using the same procedure. Conserved motifs were identified using MEME v5.3.3 (Bailey et al. 2015). Phylogenetic trees and conserved domain architectures were visualized and edited using the iTOL platform (https://itol.embl.de/). To assess selection pressures, pairwise non-synonymous to synonymous substitution rate ratios (*Ka/Ks*) were calculated using *KaKs_Calculator* and *ParaAT2.0* with default parameter, based on aligned ARF ortholog sequences from the 76 barley genotypes. Statistical significance of *Ka/Ks* differences between groups (e.g., wild vs. domesticated accessions) was tested using a two-tailed Student’s *t*-test. Tajima’s *D* values were calculated by MEGA12 and MEGA-cc software using default parameters. Visualization of Tajima’s *D* and *Ka/Ks* was performed by *R*.

### Transcriptome data processing and co-expression analysis

Raw transcriptome data from five tissues—caryopsis, coleoptile, inflorescence, root, and shoot—across 20 barley cultivars were retrieved from the recently published barley pan-transcriptome dataset (accession: PRJEB64639). Gene expression matrices (counts and TPM) were obtained from original publication (Guo et al. 2025a), where reads were mapped using STAR. Initially, quantification using Kallisto v0.46.1 (Bray et al. 2016) was also performed, and expression trends were found to be highly consistent with original data. For all downstream analyses, the STAR-based matrices were used to ensure methodological consistency. To minimize genotype-specific effects in the functional analysis of ARF genes, genotype-corrected residual expression values (${\mathrm{Residual}}_{G}$) were calculated (see below) by *R* and used for downstream analyses. Weighted gene co-expression network analysis (WGCNA) was performed using the *WGCNA* *R* package with default parameters and a correlation threshold of 0.7. Gene ontology (GO) enrichment was conducted using *clusterprofiler* v3.2.1 (Yu et al. 2012), with GO annotations sourced from the IPK barley database (https://galaxy-web.ipk-gatersleben.de/). Single-cell expression patterns of *ARF* genes were extracted from a recently published barley inflorescence single-cell transcriptomic resource BARVISTA (https://www.plabipd.de/projects/hannah_demo/BARVISTA/tissue_no_download.html) (Demesa-Arevalo et al. 2025).

### Proportion of variance explained (PVE) and residual calculation

To quantify the contribution of genotype and tissue to gene expression variation, a linear mixed model (LMM) was fitted for each ARF gene using the following equation:

$$y=\mu +G+T+\varepsilon$$

Where $y$ represents the expression value (TPM), $G \sim N\left( {0,\sigma_{G}^{2} } \right)$ is the random genotype effect, $T \sim N\left( {0,\sigma_{T}^{2} } \right)$ is the tissue effect, and $\varepsilon$ is the residual error. The proportion of variance explained by genotype and tissue was calculated as:

$${\mathrm{PVE}}_{G} = \frac{{\sigma_{G}^{2} }}{{\sigma_{G}^{2} + \sigma_{T}^{2} + \sigma_{\varepsilon }^{2} }}, {\mathrm{PVE}}_{T} = \frac{{\sigma_{T}^{2} }}{{\sigma_{G}^{2} + \sigma_{T}^{2} + \sigma_{\varepsilon }^{2} }}$$

Genotype- and tissue-corrected residuals were then extracted using:

$${\mathrm{Residual}}_{G} = y - \hat{\mu } - \hat{T}, {\mathrm{Residual}}_{T} = y - \hat{\mu } - \hat{G}$$

All calculations were performed in *R* using *lme4* package (Bates et al. 2015).

### TE identification and *cis*-eQTL analysis

TEs were identified in 76 barley genomes using *panHiTE* (Hu et al. 2025) with default parameters and further confirmed using TE annotations from the IPK panBarlex database (https://panbarlex.ipk-gatersleben.de/#). Only high-confidence TEs present in both of datasets were retained. To determine TE insertions near ARF genes, 100 kb flanking regions were analyzed using *bedtools*. For cis-eQTL analysis, 1 Mb upstream and downstream of each ARF gene was defined as the cis-regulatory region. ${\mathrm{Residual}}_{T}$ values were used as gene expression input in *MatrixEQTL* v2.3 (Shabalin 2012) under default parameters.

### Selective sweep detection

To explore potential domestication-related selection signals, raw genomic data were obtained from a recent barley population study (Guo et al. 2025b). All SNPs within a 1 Mb window flanking each ARF gene were analyzed to compare genotypic diversity among different accessions. Genomic regions were extracted using *bcftools* v1.15.1, and downstream processing and visualization were carried out using Perl under the guidance of the original study’s first author (Guo et al. 2025b).

### Variant calling and amino acid substitution prediction

Whole-genome assemblies of 76 barley accessions were fragmented into 1 kb pseudo-reads using *seqkit* v0.9.1 (Shen et al. 2016) to simulate FASTQ reads (Fig. S4). Reference genome is Morex V2. Mapping and variant calling was performed using *BWA* v0.7.17 (Li 2013), *SAMtools* v1.16.1(Li et al. 2009), and *BCFtools* v1.15.1. SNPs and InDels were annotated using *SnpEff* (Cingolani et al. 2012). All operations used default parameters. Summary of variation is shown in Fig. S4, and final vcf files could be found in the data availability part. Amino acid substitutions in ARF proteins were predicted with *PPVED* v1.0 (Gou et al. 2022). Correlation analysis between substitution scores and domestication degrees was calculated using Pearson’s correlation coefficient in *R*.

### Genome-wide association study (GWAS) and haplotype analysis

GWAS was performed to identify genomic regions associated with 16 agronomic traits (awn length; culm dry weight; fertility rate; final spikelet number; grain area; grain length; grain number per spikelet; grain weight; grain width; heading date; plant height; potential spikelet number; pre-anthesis tip degeneration; spike length; spike weight; and thousand kernels weight). Population was from previous published researches and all phenotypic data are collected at IPK Gatersleben in 2018, 2019 and 2020 (Kamal et al. 2022). Total 442 representative genotypes were used in this study; population structure was fixed by using *PLINK* v1.90b6.9 (Purcell et al. 2007). Some parts of the phenotypic data have been published in previous work (Huang et al. 2023; Kamal et al. 2022). After quality filtering, 33,138,758 SNPs (minor allele frequency > 0.1; missing rate < 10%) were retained. GWAS was conducted using *PLINK* v1.90b6.9 and *GEMMA* v0.98.5 (Team et al. 2024) with linear mixed model (LMM). All analyses used default parameters. Haplotype blocks within a 1 Mb flanking region of each ARF gene were identified to assess local linkage disequilibrium (LD) structure by using pipeline available on GitHub (https://github.com/TKNsama/R). Since some phenotypic data are unpublished, only trait-associated SNPs proximal to *ARF* loci are reported here.

### dCAPS marker design and validation

To genotype the functional HvARF3 R475G variant, a dCAPS marker was developed targeting a SNP located at chr3H:494973445 (based on the MorexV2 reference genome). Primers (Supplementary data 5) were designed using the online tools dCAPS Finder 2.0 and Primer3, incorporating a mismatch to generate a specific restriction site for the enzyme *DdeI* (New England Biolabs Inc). PCR amplification was carried out using *Taq* DNA Polymerase (Qiagen, Cat. No. 201203) following the manufacturer’s recommended protocol. Amplified products were subsequently digested with *DdeI* and separated on a 2.5% agarose gel.

## Results

### Identification and validation of *ARF* genes and their PAVs/CNVs across 76 barley pan-genome assemblies

To comprehensively catalog *ARF*-coding genes in barley, we employed a domain-based gene discovery approach by integrating three major protein family databases—Pfam (PF02362, PF06507, and PF02309), ProSiteProfiles (PS50863 and PS51745), and PANTHER (PTHR31384) — using the recently released barley pan-genome v2 database (BPGv2) (Jayakodi et al. 2024). All candidate sequences were further validated against the PanBARLEX online resource (https://panbarlex.ipk-gatersleben.de/#) to ensure annotation accuracy. Given that the conventional reference genome (Morex) lacks 2 *ARFs* due to genotype-specific presence/absence variations (PAVs), we selected the European modern spring barley cultivar Barke for gene nomenclature. Barke harbors all identified *ARF*-coding genes and has been extensively characterized at the transcriptomic level (Coulter et al. 2022; Guo et al. 2025a). To maintain cross-species consistency, gene names were assigned based on the wheat gene nomenclature system (Boden et al. 2023) and aligned with orthologous ARF genes in rice (Wang et al. 2007).

**Fig. 1:**
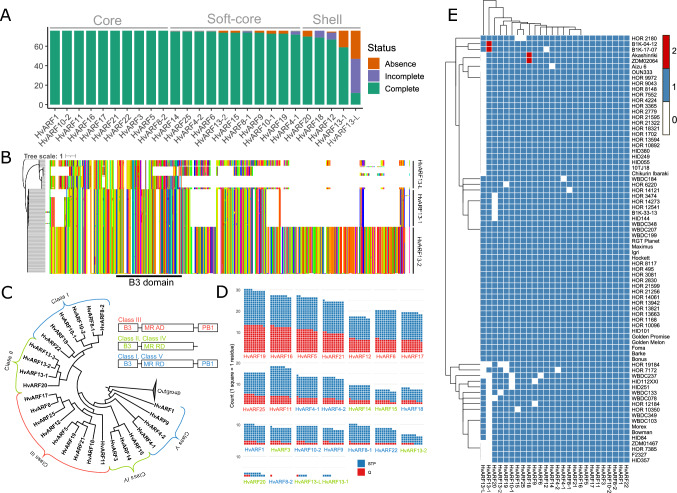
Identification and characterization of *ARF* genes in the barley pan-genome. **A** Distribution of PAVs for each *ARF* gene across 76 barley accessions. Genes were categorized as core (present in all 76 genomes), soft-core (present in ≥95% of genomes), or shell (present in 10–95% of genomes). **B** Amino acid sequence alignment of HvARF13-L, HvARF13-1, and HvARF13-2. Different colors represent different amino acid residuals. White indicates missing. **C** Phylogenetic tree of *ARF* genes in the cv. Barke, classified into five classes with different gene structure (upper right corner). B3, B3 DNA-binding domain; MR, middle region; AD, activation domain; RD, repression domain; PB1, Phox and Bem1. **D** Comparison of Q residual counts versus S/T/P residuals counts in the middle regions (MR) of ARF proteins in cv. Barke. Every square indicates one corresponding amino acid residual. **E** Heatmap showing the presence or absence of each *ARF* gene across the 76 barley genotypes

Compared to previous single-genome based analyses (Tombuloglu 2019), our pan-genomic approach uncovered approximately 30% more *ARF* genes (S1 table), underscoring the power of multi-genotype analysis to capture greater gene diversity. We retained 26 full-length ARFs that contained at least one conserved ARF-related domain in ≥10% of the accessions. This filter was only used to remove misannotated genes that completely lack ARF domains, not to exclude lineage-specific or rarely present ARFs. Thus, all correct ARF genes were included in the downstream analyses. Based on their frequency across the 76 barley genomes, these genes were classified into core (100%), soft-core (95% - 100%), and shell (15–95%) categories following established dispensability thresholds (Marroni et al. 2014) (Fig 1A, E). Notably, approximately ~80.7% of ARFs belonged to the core or soft-core categories, being present in 76 or 75 genomes, respectively. This high evolutionary conservation highlights the essential roles of ARFs for barley development and fitness.

An exception was the HvARF13 clade, which exhibited extensive structural and presence variation. While *HvARF13-2* was highly conserved (found in 74 genotypes), *HvARF13-L* and *HvARF13-1* were present in only 47 and 59 accessions, respectively. Although HvARF13-L and HvARF13-1 are shorter than HvARF13-2, all three retain an intact B3 DNA-binding domain in at least some genotypes and are expressed at transcriptome level, suggesting potential functionality (Fig. 1B). This structural variation reflects dynamic gene duplication events and copy number variations (CNVs) within the barley ARF family. To validate these PAVs, we extracted 5–10 kb flanking genomic sequences and performed local alignment. A 479 bp deletion was identified at the 3′ end of *HvARF13-1* in several genotypes, including Morex (Fig. S1B), likely explaining its absence in previous annotations. A larger deletion was detected for *HvARF13-L* in some accessions, leading to complete gene loss (Fig. S1A). Additionally, a truncated gene (HORVU.BARKE.PROJ.7HG00820280) lacking conserved domains was likely misannotated and was excluded after sequence and domain verification.

To explore functional implications of ARF protein diversity, we examined the composition of the middle region (the region between DBD domain and PB1 domain) (Cancé et al. 2022) in each Barke ARF protein, combining domain annotation with phylogenetic analysis using rice homologs (Fig. 1C). We specifically quantified glutamine (Q) and serine/threonine/proline (STP) residue enrichment, which are indicative of functional differentiation (Chandler 2016; Shen et al. 2010). Based on the phylogenetic tree and gene structure, ARFs with Q-rich middle regions were classified as Class III (typically transcriptional activators; e.g., *HvARF19*), whereas those enriched in STP residues were categorized as Class II/Class IV (without PB1 domain) or Class I/Class V (with PB1 domain) (transcriptional repressors; e.g., *HvARF1*) (Fig. 1D). Based on the results of domain identification and structural analyses, in total 26 ARF genes were used for subsequent analyses after removing misannotated genes.

### Phylogenetic, Tajima’s D, and *Ka*/*Ks* analyses reveal evolutionary dynamics and selection pressures on ARF proteins in barley

**Fig. 2:**
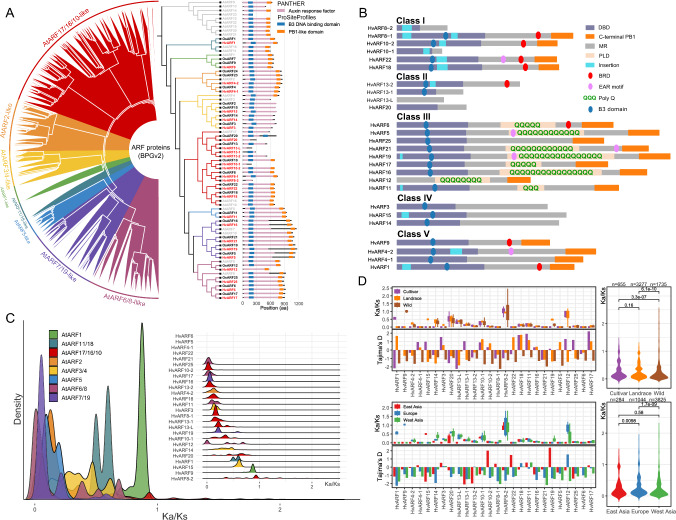
Phylogenetic relationships, protein structure, and *Ka*/*Ks* analysis of ARF proteins. **A** Phylogenetic tree of ARFs identified from 76 barley genotypes (left) and a comparative tree including ARF proteins from barley (cv. Barke), rice and *Arabidopsis* (right). Conserved protein domains of ARF proteins from Barke, rice and *Arabidopsis* are shown to the right of the phylogenetic tree. Based on Arabidopsis ARF genes, different color indicates different clades. **B** Schematic organization of ARF-MR domains in barley cv. Barke. DBD, DNA-binding domain containing the B3 domain and nearby dimerization domains; MR, middle region; PLD, prion-like domain; EAR motif, putative EAR motif characterized by the short sequence LxLxL/DLNxxP; BRD, B3 repression domain; B3 domain, the core sequence of DBD domain; Poly Q, enrich Q amino acid residuals. **C** Distribution density of *Ka/Ks* values for each ARF protein (upper right corner) and the overall *Ka/Ks* distribution across the ARF protein family (lower left corner). **D** *Ka/Ks* ratios and Tajima’s *D* values of individual ARF proteins, including comparisons among cultivars, landraces and wild barley, as well as among geographic groups: East Asia, Europe, and West Asia. *Ka/Ks* of ARFs with extremely low *Ks* have been shown as 0

To elucidate the evolutionary relationships within the ARF gene family in barley, we performed a comprehensive phylogenetic analysis based on full-length ARF protein sequences derived from 76 barley genotypes. Despite extensive sequence variation among accessions, all ARF proteins could be reliably assigned to their respective clades, consistent with ARF classifications established in other plant species (Wang et al. 2007) (Fig. 2A). Comparative genome analyses across *Arabidopsis*, rice, and barley revealed substantial changes in ARF copy number, with both gene losses and expansions observed. Notably, several *Arabidopsis* ARFs, including *AtARF9*, *AtARF13*, *AtARF14*, *AtARF23*, *AtARF12*, *AtARF22*, *AtARF15*, *AtARF10*, *AtARF20*, and *AtARF21*, were completely absent in both rice and barley (Fig. 2A), suggesting lineage-specific gene loss events following the divergence of grasses and dicots (Aravind et al. 2000; Finet et al. 2013). This pattern likely reflects evolutionary streamlining of the ARF repertoires in grasses and possibly the adoption of distinct auxin signaling strategies (McSteen 2010).

Interestingly, the AtARF5 clade retained a single-copy ortholog across all three species tested here, indicative of strong purifying selection and a critical, possibly irreplaceable, role in plant development. In contrast, barley showed expanded *ARF* families compared to rice, particularly in *HvARF4*, *HvARF8*, *HvARF10*, and *HvARF13*. Among these, *HvARF4-1* and *HvARF4-2* represent a rare example of full-length gene duplication, with both paralogs retaining all key functional domains, including the B3 DNA-binding domain and PB1-like domain (Fig. 2A, B). Other duplicated genes, such as members of the HvARF13 clade, often exhibited partial structures lacking one or more conserved domains, suggesting potential subfunctionalization or neofunctionalization. Notable, most of the observed barley-specific ARF duplications clustered within the AtARF17/16/10-like clade, which includes *AtARF10*, *AtARF16*, *AtARF17*, and *OsARF18*—a group known to function as negative regulators of plant development and seed germination (Huang et al. 2016; Liu et al. 2007; Liu et al. 2013; Mallory et al. 2005). The increasing copy number of this clade from *Arabidopsis* to rice and barley suggests an adaptive expansion, potentially driven by environmental selection pressures or functional diversification. To investigate the variation among ARF protein in barley, all ARF protein sequences were analyzed and are presented in a schematic diagram (Fig. 2B) (Cancé et al. 2022). The DBD domain is relatively conserved in class I ARFs, whereas almost all class III ARFs contain a short insertion within this region, suggesting greater structural stability and conservation in class I. Notably, HvARF25, although belonging to class I, has a relatively high content of glutamine (Q) residues but does not form a poly-Q structure. Most class III ARFs possess the B3 repression domain (BRD, motif pattern: R/KLFG) (Choi et al. 2018), a characteristic indicator of repressor function. However, HvARF4-1 and HvARF4-2 are exceptions: instead of the canonical R/KLFG motif, each contain a single amino acid substitution that produces a modified KIFG motif, potentially indicating a functional divergence.

To assess selection constraints acting on ARF proteins, we calculated the ratio of non-synonymous (*Ka*) to synonymous (*Ks*) substitution rates (*Ka/Ks*) across different barley populations. *Ka/Ks* values below, equal to, or above 1 are generally interpreted as indicative of purifying, neutral, or positive selections, respectively (Kimura 1985). Considering the high sequence homology among alleles within a species, we excluded nearly identical pairs (Ks ≤ 0.01) and retained only moderately diverged alleles (Ks > 0.01) to better capture genuine evolutionary or purifying selection signals rather than intraspecies sequence conservation (HvARF6, HvARF5, HvARF4-1, HvARF22, and HvARF21 were filtered with extremely low Ks). Overall, most ARF proteins exhibited *Ka/Ks* ratios well below 1, indicating strong purifying selection acting to conserve gene function across genotypes (Fig. 2C). This evolutionary constraint underscores the essential regulatory roles of ARF proteins during barley development and adaptation. However, *HvARF8-2* was a notable exception, with *Ka/Ks* ratios frequently exceeding 1. The lack of a B3 domain in this gene may have relaxed functional constraints, permitting rapid evolution under natural or selective breeding. This gene may represent an example of lineage-specific divergence or a recently neofunctionalized paralog. Further comparison of *Ka/Ks* ratios among wild barley, landraces, and modern cultivars revealed a statistically significant decline between wild accessions and cultivars/landraces, indicative of intensified purifying selection during domestication and breeding. A representative example is *HvARF9*, which shows a *Ka*/*Ks* ratio close to 1 in wild barley but nearly 0 in landrace and cultivar groups, indicating that this gene became highly conserved after domestication. A similar trend was observed across geographic regions: accessions from East Asia exhibited lower *Ka/Ks* values than those from Europe and West Asia (Fig. 2D), suggesting differential selection pressures linked to regional adaptation. For Tajima’s *D*, cultivars and landraces generally exhibited higher values than wild barley, suggesting potential effects of artificial selection or population bottlenecks during domestication. Notably, for HvARF10-2, HvARF22, and HvARF19, populations from East Asia displayed higher *D* values compared to those from other regions, indicating that selective breeding, historical bottlenecks, or subpopulation admixture may have influenced the genetic variation of these genes in this region.

Interestingly, *HvARF12* deviated from this general pattern, showing elevated *Ka/Ks* ratios in cultivars and landrace compared to wild accessions. Its rice ortholog, *OsARF12*, is functionally redundant with *OsARF17* and *OsARF25* and has been implicated in auxin-mediated regulation of tiller angle, as well as in responses to viral infection and disease resistance (Li et al. 2020) (Zhao et al. 2022). These findings raise the possibility that *HvARF12* has undergone adaptive evolution, possibly driven by trade-offs between agronomic performance and stress tolerance.

Taken together, these results indicate diverse evolutionary dynamics within the *ARF* family in barley, possibly reflecting the combined effects of natural selection, domestication, and breeding history. The integration of phylogenetic, structural, Tajima’s *D,* and *Ka/Ks* analyses provides a framework for understanding functional divergence and adaptation of *ARF* genes in cereal crops.

### TE identification, classification, and their regulatory influence on *ARF* gene expression

Transposable elements (TEs) are mobile genetic elements capable of within the genome, frequently inducing mutations or altering gene expression. Representing a substantial fraction of plant genomes, TEs have been recognized as major contributors to genome evolution, structural variation, and transcriptional regulation (Bennetzen and Wang 2014; Hirsch and Springer 2017; Lisch 2013). To comprehensively investigate the potential cis-regulatory effects of TEs on ARF gene expression, we followed previous studies (Schmitz et al. 2022) and examined TE insertions within 100 kb upstream and downstream of all ARF genes across 76 barley accessions, using a high-confidence TE annotation dataset.

**Fig. 3:**
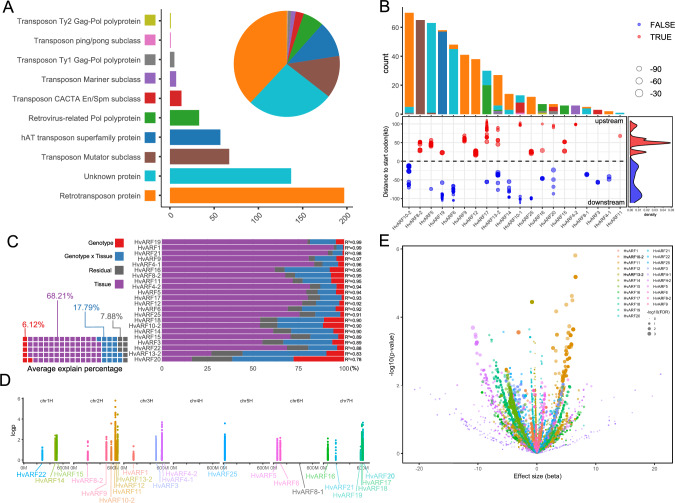
Classification of TEs and eQTL analysis of ARF genes. **A** Number and classification of TEs located within 100 kb flanking regions of each ARF gene across 76 barley genotypes. **B** TE types, counts, and positional distribution around ARF genes. Density plot shows the enrichment of TEs relative to ARF gene loci. **C** Proportion of expression variance explained each ARF gene by genotype, tissue, and genotype × tissue interaction. The waffle chart displays the average contribution of each factor across all genes. **D** *cis*-eQTL signals for ARF genes within a ±1 Mb region. **E** Volcano plot of eQTL-associated SNPs. The X-axis represents effect size, and the Y-axis shows − log10(*P*-value)

In total, 516 TEs were identified near ARF loci, encompassing diverse TE families, with retrotransposons constituting the predominant class (Fig. 3A). Interestingly, TE insertions were not randomly distributed: Significantly enrichment was observed within the 50 kb upstream of many *HvARF* genes. A secondary enrichment peak occurred around 20 kb upstream of several loci (Fig. 3B). Given their proximity to core promoter regions and their established roles in modulating chromatin accessibility and transcription factor binding (Hirsch and Springer 2017; Lönnig and Saedler 2002), these upstream TEs are strong candidates for modulating *ARF* gene expression via *cis*-regulatory mechanisms. The observed spatial enrichment pattern suggests non-random TE insertion or retention, possibly reflecting selection for regulatory function. To investigate the potential regulatory impact of nearby TEs, we conducted expression quantitative trait loci (eQTL) mapping (Druka et al. 2010) in a panel of 20 representative genotypes by integrating high-quality pan-genome and pan-transcriptome datasets (Guo et al. 2025a). To disentangle the complex sources of expression variation, we partitioned the variance in gene expression into components attributable to genotype, tissue, and their interaction. Among the 26 ARF genes, 22 displayed sufficient expression variability and were retained for further analysis.

Variance component analysis revealed that tissue and tissue × genotype interactions were the primary drivers of expression variation, consistent with the known tissue-specific expression profiles of *ARF* genes (Fig. 3C). To mitigate tissue effects in subsequent association analyses, we used residual expression values—obtained by regressing out tissue effects—instead of raw expression levels. Following previous studies (Pitz et al. 2025) and considering our aim to capture potential eQTL peaks associated with nearby TE insertions, *cis*-eQTLs were identified using SNPs located within a 1 Mb window flanking each ARF gene, in order to comprehensively include potential regulatory signals. As anticipated, genes with high genotype or genotype-by-tissue explained variance (PVE) — such as *HvARF20*, *HvARF13-2*, *HvARF18*, and *HvARF25*—displayed strong eQTL signals (–log₁₀P > 3) (Fig. 3D, E). Notably, *HvARF25* exhibited a distinct eQTL peak located 23–32 kb upstream of its transcriptional start site, coinciding with the position of a retrotransposon. This suggests that the TE insertion may act as a *cis*-regulatory element modulating *HvARF25* expression in a genotype-dependent manner (Fig. 3B, D).

A particularly interesting outlier was *HvARF11*, which showed the most significant eQTL signal despite having low genotype and genotype-by-tissue PVE, and lacking annotated TEs within its vicinity (Fig. 3 D, E). This implies the presence of a potent, localized regulatory variant—potentially an unannotated TE insertion, structural variation, or epigenetic modification—contributing substantially to *HvARF11* expression, independent of broader genomic background effects. Overall, TE located near *ARF* genes are likely to play a regulatory role contributing to genotype-specific expression differences.

### Pan-transcriptomic analysis reveals tissue-specific expression and functional networks of ARF genes

To elucidate the functional roles and tissue-specific regulatory dynamics of *ARF* genes in barley, we integrated population-scale pan-genomic (Jayakodi et al. 2024) and pan-transcriptomic (Guo et al. 2025a) datasets. A total of 22 ARF genes with consistently detectable expression across five major tissues in 20 representative genotypes were retained for downstream analyses, minimizing noise from low-abundance transcripts.

**Fig. 4:**
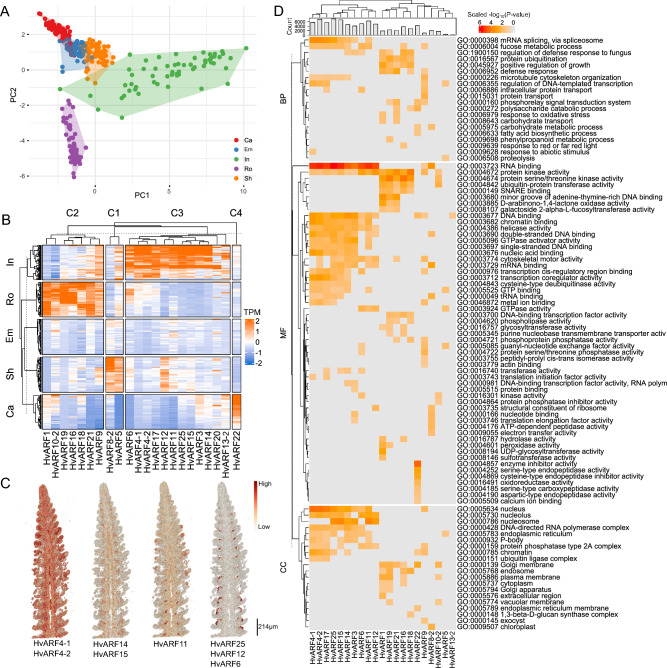
Pan-transcriptome analysis of each *ARF* gene expression. **A** PCA of ARF gene expression across all genotype-tissue combinations. PC1 and PC2 explain 41.5% and 24.1% of the total variance, respectively. Colors indicate tissue types; shaded areas highlight genotype-dependent variation within each tissue. Ca, caryopsis; Em, embryo; In, inflorescence; Ro, root; Sh, shoot. **B** Summary heatmap showing expression levels of ARF genes across five tissues and 20 genotypes. C1-C4, cluster 1 to cluster 4. **C** Single-cell transcriptomic visualization of *ARF* gene expression in barley inflorescence (Demesa-Arevalo et al. 2025). Color intensity represents relative expression level; scale bar = 214 µm. **D** GO enrichment analysis of gene co-expression modules associated with each *ARF* gene (co-expression threshold ≥ 0.7). Color scale indicates − log_10_(*P*-value) normalized by *Z*-score

ARF genes displayed pronounced tissue-specific expression patterns, with the inflorescence exhibiting the highest genotype-dependent expression variability, followed by roots (Fig. 4A). Notably, 12 of the 22 ARF genes were highly and specifically expressed in developing inflorescences at Waddington stages W6–W7 (Waddington et al. 1983), a critical window for spikelet and floret meristem differentiation. Seven genes showed peak expression in seedling roots, while *HvARF2* and *HvARF6* were predominantly expressed in shoots tissues (Fig. 4B). *HvARF22* exhibited unique expression in caryopsis, and no ARF genes demonstrated tissue specificity in embryonic structures such as the embryo, mesocotyl, or seminal root. To further resolve the spatial expression dynamics within inflorescence tissue, we utilized a high-resolution single-cell RNA-seq atlas of barley inflorescence (Morex) (Demesa-Arevalo et al. 2025). This analysis uncovered cell-type-specific expression patterns for individual ARF genes (Fig. 4C). *HvARF4-1* and *HvARF4-2* were broadly expressed across diverse cell types, indicating general developmental roles. In contrast, *HvARF14*, *HvARF15*, and *HvARF11* were enriched in vascular-associated tissues, with *HvARF11* being specifically expressed during the transition from triple-spikelet meristem to floret meristem—an essential developmental checkpoint. Additionally, *HvARF6*, *HvARF12*, and *HvARF25* were predominantly expressed in the adaxial epidermis, suggesting possible roles in organ polarity or boundary formation. These temporally and spatially resolved expression profiles support the hypothesis that ARF genes operate in multiple, partially distinct regulatory programs during inflorescence development.

To further explore the potential regulatory networks associated with those ARF gene, we conducted a weighted gene co-expression network analysis (WGCNA). Residual expression values (adjusted for genotypic effects) were used as input to focus on tissue-specific transcriptional relationships. For each ARF gene, co-expressed gene modules were identified, and gene ontology (GO) enrichment analysis was performed (Fig. 4D). As expected for transcription factors, most *HvARF*-associated modules were enriched in functions related to RNA/DNA binding and transcriptional regulation. Notably, *HvARF3*, *HvARF14*, and *HvARF15* were co-expressed with genes involved in fungal defense responses. This observation is consistent with the functional identification of their rice orthologs (*OsARF3*, *OsARF3a*, and *OsARF3b*), which have been reported to modulate disease resistance pathways.

Overall, our discovery indicates that *ARF* genes exhibit tissue and cell-type-specific expression patterns and are involved in distinct regulatory modules that govern a range of biological processes—from developmental patterning in inflorescence to biotic stress responses. These results insight a foundational framework for future functional studies and genetic manipulation strategies for optimizing inflorescence architecture or resistance in barley.

### Pan-genome-based multi-omics analysis identifies HvARF3 as a key target of selection during barley breeding

**Fig. 5:**
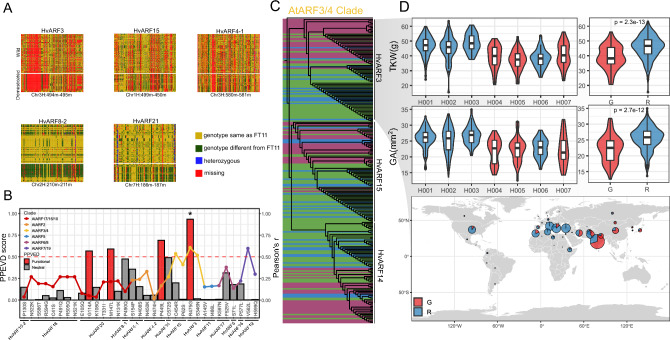
Germplasm analysis of ARF genes and haplotype analysis of HvARF3. **A** SNP matrix showing putative selective sweep regions (1 Mb) for five ARF genes. Different colors indicate nucleotide polymorphisms at each site across genotypes. **B** Bar plot showing PPVED-predicted functional impact scores (left Y-axis) and Pearson’s correlation coefficients (right Y-axis) for amino acid substitutions across ARF proteins. Clade is same as in the phylogenetic tree in Fig. 2A. **C** Phylogenetic trees of HvARF3, HvARF14, and HvARF15, with wild (purple), landrace (blue), and cultivar (green) accessions indicated. Outgroup is *AtARF3*. **D** Haplotype analysis in terms of grain trait (upper part) and geographic distribution of HvARF3 (lower part) across the barley diversity panel. TKW, thousand kernel weight; GA, grain area

To investigate the role of *ARF* gene variation in barley domestication and improvement, we performed selective sweep analyses alongside functional predictions of amino acid (AA) substitutions. Selection signals were examined within genomic regions flanking each *ARF* gene using a population panel comprising wild barley, landraces, and modern cultivars (Fig. S3). Among the 26 *ARF* loci, five—*HvARF3*, *HvARF8-2*, *HvARF21*, *HvARF15*, and *HvARF4-1*—exhibited strong selection signatures, with marked allelic differentiation between wild and domesticated accessions (Fig. 5A). These findings suggest that these loci may have undergone positive selection due to their contributions to agronomic traits favored during domestication and breeding.

To assess the potential functional relevance of naturally occurring amino acid substitutions, we predicted the effects of all non-synonymous variants (allele frequency > 0.1) using the plant protein variation effect detector (PPVED) (Gou et al. 2022). Pearson’s correlation analyses between amino acid variants and domestication scores identified several candidate residues under selection (Fig. 5B). Four variants had PPVED scores > 0.5, indicating a high probability of functional impact, while the remaining substitutions were predicted to be functionally neutral. The most notable protein variant was a non-synonymous substitution in HvARF3 (R475G), which had the highest predicted functional impact across the ARF protein family. HvARF3, along with HvARF14 and HvARF15, belongs to the AtARF3/4 orthologous clade. All three members of this subclade exhibited strong selection signals despite being located on different chromosomes, suggesting that similar selective pressures may have acted on this regulatory lineage (Fig. 5B). Phylogenetic analysis of ARF proteins across the 76 barley accessions further supported this observation. While most ARF clades contained a mixture of wild and domesticated accessions, HvARF3, HvARF14, and HvARF15 formed lineage-specific clusters corresponding to domestication groups (Fig. 5C). This convergence of selective sweep signals, phylogenetic divergence, and predicted functional variant highlights the AtARF3/4 subclade—particular HvARF3—as a key target of directional selection linked to agronomic adaptation.

To investigate the phenotypic consequences of *ARF* gene variation, we performed genome-wide association studies (GWAS) focused on key yield components, particularly grain size and thousand kernel weight (TKW). SNPs within 1 Mb of each *ARF* gene were analyzed using a significance threshold of –log_10_(*P*) > 2. While most associations showed modest effect sizes, consistent with the pleiotropic and conserved roles of *ARF* genes, we observed consistent association peaks near *HvARF3* (8.89 kb from peak) and *HvARF4-1* (2.41 kb from peak), particularly for TKW and grain size (Fig. S2 C, Table S4). To validate the association of *HvARF3* with grain traits, we performed haplotype analysis based on SNPs within the gene body and its flanking regulatory regions. Seven major haplotypes were identified and could be classified into two distinct groups based on the R475G substitution: the “R475” allele, predominantly present in European cultivars, and the “G475” allele, more frequently observed in Asian accessions. Accessions harboring the R475 allele exhibited significantly higher grain area (GA) and TKW, two key traits under strong selection during modern barley breeding (Figure 5D). Notably, this positive association between the R475 allele and increased grain size and weight remained significant even after correcting for the *nud* locus (responsible for the naked vs. hulled grain phenotype) (Taketa et al. 2008), indicating that allelic variation at *HvARF3* contributes to grain trait variation independently of hull type (Fig. S2B).

To facilitate marker-assisted selection, a derived cleaved amplified polymorphic sequence (dCAPS) marker was developed to discriminate between the R and G alleles at the HvARF3 R475G site (Fig. S5). Collectively, this result provides indirect but convincing evidence that *HvARF3* regulates grain traits and has undergone selection during barley improvement.

## Discussion

In this study, we present a method of gene family analysis by utilizing abundant, publicly available genomic and transcriptomic resources to comprehensively investigate the *ARF* gene family in barley. Through the integration of multi-omics datasets, we performed a series of systematic analyses including PAV detection, evolutionary dynamics, TE-mediated eQTL mapping, transcriptome analysis, and domestication signal identification. A summary of *ARF* genes in barley has been created by using different data source and methods, which provide insights and clues for future cloning and analysis. Those analyses, which were previously impossible with a single reference genome, demonstrate the advantage of pan-genome, pan-transcriptome, and resequencing data in mining gene family complexity across diverse genotypes in barley.

Recent advances in barley multi-omics resources, including high-resolution pan-genomes and population-wide transcriptomes (Guo et al. 2025a), offer unprecedented opportunities for gene family research. However, the challenge lies in effective data integration and interpretation. Our study demonstrates that combining these layers enables the discovery of functional alleles and regulatory mechanisms otherwise inaccessible. For instance, the identification of HvARF3^R475G^ as a putative breeding-related variant associated with increased grain size and TKW underscores the value of population-scale pan-genomic approaches. Conventional gene family studies relying on single genomes often fail to capture intraspecies diversity—especially in crops like barley with global distribution and rich germplasm pools. Pan-genomics strategies, in contrast, enable the identification of both structural and nucleotide-level polymorphisms, accelerating trait mapping and gene discovery. Previously, genes such as *Ppd-H1* and *VRN-1* were identified through large-scale germplasm screenings (Santra et al. 2009; Turner et al. 2005). With the availability of pan-genome datasets, researchers can now achieve earlier and more targeted discovery of genes and genetic variations associated with key traits. In this study, we revealed geographic stratification of HvARF3 haplotypes—paralleling patterns observed in well-characterized flowering regulators like *Ppd-H1* (Lister et al. 2009)—highlighting possible regional adaptation and selective breeding.

The *ARF* gene family comprises a moderate number of transcription factors with essential roles in auxin-mediated growth and development. While several *ARFs* have been functionally characterized in *Arabidopsis* and rice, their roles in barley remain underexplored. Phylogenetic analyses revealed lineage-specific expansions and contractions. Notable, the AtARF17/16/10 clade exhibited multiple duplications in barley, particularly within the *HvARF13* subgroup. *HvARF13-2* remains highly conserved, while *HvARF13-L* and *HvARF13-1* show CNVs and sequence divergence—suggesting ongoing subfunctionalization or neofunctionalization (Fig. 1B). These variations are more frequent in wild and landrace accessions, implying adaptive relevance in non-domesticated environments. Comparative genomics also revealed notable differences in *ARF* gene retention and loss. For example, rice contains two paralogs, *OsARF23* and *OsARF24* (formerly OsARF1), involved in grain formation via *rice morphology determinant (RMD)* regulation (Li et al. 2014; Waller et al. 2002). These genes have no direct orthologs in barley (Fig. 2A), indicating species-specific divergence. Conversely, barley harbors a duplicated *OsARF4* ortholog pair—*HvARF4-1* and *HvARF4-2*—both retaining full-length domain structures. Given *OsARF4’s* known role in negatively regulating grain size in rice (Hu et al. 2018), its barley counterparts represent strong candidates for functional studies.

Despite previous findings that several rice *ARF* genes (e.g., *OsARF3*, *OsARF4*, *OsARF6*, *OsARF14*, and *OsARF15*) are associated with grain-related traits (Gu et al. 2023; Hu et al. 2018; Qiao et al. 2021), most *ARF* genes in barley do not show direct expression in the caryopsis (Fig. 4B). Instead, their strong expression in inflorescence tissues suggests that these *ARF* genes may regulate grain development indirectly by modulating early reproductive organogenesis and floral architecture, which likely affects final grain size or weight. Another example is *HvARF3*, the selected gene during breeding, it shows high gene expression levels in the developing inflorescence while no expression in grain. In the single-cell transcriptome dataset of barley inflorescences, *HvARF3* is specifically expressed in dividing cells, suggesting a potential role in regulating cell division and meristem maintenance during early stages of inflorescence development. But according to GWAS haplotype analysis and performance of its ortholog *OsARF3*, it most likely affects grain size and grain traits, too. Interestingly, the favorable haplotypes of *HvARF3* are rare in Asian barley accessions. This may be attributable to a regional genetic bottleneck or local adaptation, which limited the introgression of the advantageous haplotype. Alternatively, certain *HvARF3* alleles may exhibit functional trade-offs similar to its rice ortholog *OsARF3*, which has been shown to mediate a balance between heat tolerance and pathogen resistance (Gu et al. 2023). Furthermore, The *Ka/Ks* ratios of *HvARF3* varied across regions, implying that different populations experienced distinct levels of purifying selection under varying environmental conditions. Thus, environmental difference between European and Asian growth conditions could have shaped regional selection pressures acting on different *HvARF3* variants and reflect different breeding strategy. However, although multiple analyses suggest that HvARF3 plays an important role, its precise function still requires experimental validation.

In conclusion, our integrative analysis highlights the critical role of *ARF* genes in barley development and demonstrates the utility of multi-omics strategies in gene family research. By resolving patterns of structural variation, expression regulation, and selection, we primarily identify promising candidates like *HvARF3* for targeted breeding. These findings contribute not only to our understanding of auxin signaling but also to practical efforts aimed at improving grain yield and adaptability in barley. The dCAPS marker developed for the HvARF3^R475^ variant will facilitate marker-assisted selection in future breeding programs.

## Supplementary Information

Below is the link to the electronic supplementary material.

Supplementary file1 (PDF 93 KB)

Supplementary file2 (PDF 1086 KB)

Supplementary file3 (PDF 6379 KB)

Supplementary file4 (PDF 620 KB)

Supplementary file5 (PNG 594 KB)

Supplementary file6 (XLSX 48 KB)

## Data Availability

Seventy-six pan-genome sequence and annotation data are from previous publication (Jayakodi et al. 2024); Genotypic data of wild barley/domesticated barley are from previous publication (Guo et al. 2025b); Genotypic data of GWAS will be released in previous and upcoming publication (Huang et al. 2023; Kamal et al. 2022); some phenotypic data (FSN, GY, HD, MYP, PH, PSN, and SA) are from previous publication (Kamal et al. 2022), while rest of them will be released in upcoming publication. The re-mapping VCF files created by this study have been deposited at Zenodo (https://zenodo.org/records/16778621).

## References

[CR1] Aravind L, Watanabe H, Lipman DJ, Koonin EV (2000) Lineage-specific loss and divergence of functionally linked genes in eukaryotes. Proc Natl Acad Sci U S A 97:11319–11324 11016957 10.1073/pnas.200346997 PMC17198

[CR2] Bailey TL, Johnson J, Grant CE, Noble WS (2015) The MEME suite. Nucleic Acids Res 43:W39–W49 25953851 10.1093/nar/gkv416 PMC4489269

[CR3] Bates D, Mächler M, Bolker B, Walker S (2015) Fitting linear mixed-effects models using lme4. J Stat Soft 67:1–48

[CR4] Bennetzen JL, Wang H (2014) The contributions of transposable elements to the structure, function, and evolution of plant genomes. Annu Rev Plant Biol 65:505–530 24579996 10.1146/annurev-arplant-050213-035811

[CR5] Boden S, McIntosh R, Uauy C, Krattinger SG, Dubcovsky J, Rogers WJ, Xia X, Badaeva E, Bentley A, Brown-Guedira G (2023) Updated guidelines for gene nomenclature in wheat. Theor Appl Genet 136:72 36952017 10.1007/s00122-023-04253-w PMC10036449

[CR6] Bray NL, Pimentel H, Melsted P, Pachter L (2016) Near-optimal probabilistic RNA-seq quantification. Nat Biotechnol 34:525–527 27043002 10.1038/nbt.3519

[CR7] Camacho C, Coulouris G, Avagyan V, Ma N, Papadopoulos J, Bealer K, Madden TL (2009) BLAST+: architecture and applications. BMC Bioinformatics 10:1–9 20003500 10.1186/1471-2105-10-421 PMC2803857

[CR8] Cancé C, Martin-Arevalillo R, Boubekeur K, Dumas R (2022) Auxin response factors are keys to the many auxin doors. New Phytol 235:402–419 35434800 10.1111/nph.18159

[CR9] Chandler JW (2016) Auxin response factors. Plant Cell Environ 39:1014–1028 26487015 10.1111/pce.12662

[CR10] Choi H-S, Seo M, Cho H-T (2018) Two TPL-binding motifs of ARF2 are involved in repression of auxin responses. Front Plant Sci 9:372 29619039 10.3389/fpls.2018.00372 PMC5871684

[CR11] Cingolani P, Platts A, Wang LL, Coon M, Nguyen T, Wang L, Land SJ, Lu X, Ruden DM (2012) A program for annotating and predicting the effects of single nucleotide polymorphisms, SnpEff: SNPs in the genome of *Drosophila melanogaster* strain w1118; iso-2; iso-3. Fly 6:80–92 22728672 10.4161/fly.19695 PMC3679285

[CR12] Coulter M, Entizne JC, Guo W, Bayer M, Wonneberger R, Milne L, Schreiber M, Haaning A, Muehlbauer GJ, McCallum N (2022) BaRTv2: a highly resolved barley reference transcriptome for accurate transcript-specific RNA-seq quantification. Plant J 111:1183–1202 35704392 10.1111/tpj.15871 PMC9546494

[CR13] Delcher AL, Salzberg SL, Phillippy AM (2003) Using MUMmer to identify similar regions in large sequence sets. Curr Protoc Bioinformatics. 10.1002/0471250953.bi1003s00 18428693 10.1002/0471250953.bi1003s00

[CR14] Demesa-Arevalo E, Dörpholz H, Vardanega I, Maika JE, Pineda-Valentino I, Eggels S, Lautwein T, Köhrer K, Schnurbusch T, von Korff M, Usadel B, Simon R (2025) Imputation integrates single-cell and spatial gene expression data to resolve transcriptional networks in barley shoot meristem development. bioRxiv. 2025.2005.2009.653223 10.1038/s41477-025-02176-6 PMC12830361 41501532

[CR15] Druka A, Potokina E, Luo Z, Jiang N, Chen X, Kearsey M, Waugh R (2010) Expression quantitative trait loci analysis in plants. Plant Biotechnol J 8:10–27 20055957 10.1111/j.1467-7652.2009.00460.x

[CR16] Finet C, Berne-Dedieu A, Scutt CP, Marlétaz F (2013) Evolution of the ARF gene family in land plants: old domains, new tricks. Mol Biol Evol 30:45–56 22977118 10.1093/molbev/mss220

[CR17] Finn RD, Clements J, Eddy SR (2011) HMMER web server: interactive sequence similarity searching. Nucleic Acids Res 39:W29–W37 21593126 10.1093/nar/gkr367 PMC3125773

[CR18] Gao J, Zhang L, Du H, Dong Y, Zhen S, Wang C, Wang Q, Yang J, Zhang P, Zheng X (2023) An ARF24-ZmArf2 module influences kernel size in different maize haplotypes. J Integr Plant Biol 65:1767–1781 36866706 10.1111/jipb.13473

[CR19] Gidhi A, Kumar M, Mukhopadhyay K (2021) The auxin response factor gene family in wheat (*Triticum aestivum* L.): genome-wide identification, characterization and expression analyses in response to leaf rust. S Afr J Bot 140:312–325

[CR20] Gou X, Feng X, Shi H, Guo T, Xie R, Liu Y, Wang Q, Li H, Yang B, Chen L (2022) Ppved: a machine learning tool for predicting the effect of single amino acid substitution on protein function in plants. Plant Biotechnol J 20:1417–1431 35398963 10.1111/pbi.13823 PMC9241370

[CR21] Gu X, Si F, Feng Z, Li S, Liang D, Yang P, Yang C, Yan B, Tang J, Yang Y (2023) The OsSGS3-tasiRNA-OsARF3 module orchestrates abiotic-biotic stress response trade-off in rice. Nat Commun 14:4441 37488129 10.1038/s41467-023-40176-2 PMC10366173

[CR22] Guo N, Wang Y, Chen W, Tang S, An R, Wei X, Hu S, Tang S, Shao G, Jiao G (2022) Fine mapping and target gene identification of qSE4, a QTL for stigma exsertion rate in rice (*Oryza sativa* L.). Front Plant Sci 13:959859 35923872 10.3389/fpls.2022.959859 PMC9341389

[CR23] Guo W, Schreiber M, Marosi VB, Bagnaresi P, Jørgensen ME, Braune KB, Chalmers K, Chapman B, Dang V, Dockter C (2025a) A barley pan-transcriptome reveals layers of genotype-dependent transcriptional complexity. Nat Genet 57(2):441–450 39901014 10.1038/s41588-024-02069-y PMC11821519

[CR24] Guo Y, Jayakodi M, Himmelbach A, Ben-Yosef E, Davidovich U, David M, Hartmann-Shenkman A, Kislev M, Fahima T, Schuenemann VJ, Reiter E, Krause J, Steffenson BJ, Stein N, Weiss E, Mascher M (2025b) A haplotype-based evolutionary history of barley domestication. Nature 647:680–688 40993384 10.1038/s41586-025-09533-7 PMC12629985

[CR25] Hirsch CD, Springer NM (2017) Transposable element influences on gene expression in plants. Biochimica et Biophysica Acta (BBA) 1860:157–165 10.1016/j.bbagrm.2016.05.010 27235540

[CR26] Hu Z, Lu S, Wang M, He H, Sun L, Wang H, Liu X, Jiang L, Sun J, Xin X (2018) A novel QTL qTGW3 encodes the GSK3/SHAGGY-like kinase OsGSK5/OsSK41 that interacts with OsARF4 to negatively regulate grain size and weight in rice. Mol Plant 11:736–749 29567449 10.1016/j.molp.2018.03.005

[CR27] Hu K, Xu M, Wang J (2025) PanHiTE: a comprehensive and accurate pipeline for TE detection in large-scale population genomes. bioRxiv. 2025.2002.2015.638472 10.1016/j.xplc.2025.101669 PMC12983257 41376169

[CR28] Huang J, Li Z, Zhao D (2016) Deregulation of the Os miR160 target gene OsARF18 causes growth and developmental defects with an alteration of auxin signaling in rice. Sci Rep 6:29938 27444058 10.1038/srep29938 PMC4956771

[CR29] Huang Y, Kamal R, Shanmugaraj N, Rutten T, Thirulogachandar V, Zhao S, Hoffie I, Hensel G, Rajaraman J, Moya YAT, Hajirezaei MR, Himmelbach A, Poursarebani N, Lundqvist U, Kumlehn J, Stein N, von Wiren N, Mascher M, Melzer M, Schnurbusch T (2023) A molecular framework for grain number determination in barley. Sci Adv 9:eadd0324 36867700 10.1126/sciadv.add0324 PMC9984178

[CR30] Jayakodi M, Padmarasu S, Haberer G, Bonthala VS, Gundlach H, Monat C, Lux T, Kamal N, Lang D, Himmelbach A (2020) The barley pan-genome reveals the hidden legacy of mutation breeding. Nature 588:284–289 33239781 10.1038/s41586-020-2947-8 PMC7759462

[CR31] Jayakodi M, Lu Q, Pidon H, Rabanus-Wallace MT, Bayer M, Lux T, Guo Y, Jaegle B, Badea A, Bekele W (2024) Structural variation in the pangenome of wild and domesticated barley. Nature. 10.1038/s41586-024-08187-1 39537924 10.1038/s41586-024-08187-1 PMC11655362

[CR32] Jia M, Li Y, Wang Z, Tao S, Sun G, Kong X, Wang K, Ye X, Liu S, Geng S (2021) TaIAA21 represses TaARF25-mediated expression of TaERFs required for grain size and weight development in wheat. Plant J 108:1754–1767 34643010 10.1111/tpj.15541

[CR33] Kamal R, Muqaddasi QH, Zhao Y, Schnurbusch T (2022) Spikelet abortion in six-rowed barley is mainly influenced by final spikelet number, with potential spikelet number acting as a suppressor trait. J Exp Bot 73:2005–2020 34864992 10.1093/jxb/erab529

[CR34] Katoh K, Standley DM (2013) MAFFT multiple sequence alignment software version 7: improvements in performance and usability. Mol Biol Evol 30:772–780 23329690 10.1093/molbev/mst010 PMC3603318

[CR35] Kimura M (1985) The neutral theory of molecular evolution. Cambridge University Press, Cambridge

[CR36] Li H (2018) Minimap2: pairwise alignment for nucleotide sequences. Bioinformatics 34:3094–3100 29750242 10.1093/bioinformatics/bty191 PMC6137996

[CR37] Li H, Handsaker B, Wysoker A, Fennell T, Ruan J, Homer N, Marth G, Abecasis G, Durbin R (2009) The sequence alignment map/format and SAMtools. Bioinformatics 25:2078–2079 19505943 10.1093/bioinformatics/btp352 PMC2723002

[CR38] Li G, Liang W, Zhang X, Ren H, Hu J, Bennett MJ, Zhang D (2014) Rice actin-binding protein RMD is a key link in the auxin–actin regulatory loop that controls cell growth. Proc Natl Acad Sci U S A 111:10377–10382 24982173 10.1073/pnas.1401680111 PMC4104909

[CR39] Li Y, Li J, Chen Z, Wei Y, Qi Y, Wu C (2020) OsmiR167a-targeted auxin response factors modulate tiller angle via fine-tuning auxin distribution in rice. Plant Biotechnol J 18:2015–2026 32061119 10.1111/pbi.13360 PMC7540336

[CR40] Li Y, Han S, Qi Y (2023) Advances in structure and function of auxin response factor in plants. J Integr Plant Biol 65:617–632 36263892 10.1111/jipb.13392

[CR41] Li H (2013) Aligning sequence reads, clone sequences and assembly contigs with BWA-MEM. arXiv preprint arXiv:13033997

[CR42] Lisch D (2013) How important are transposons for plant evolution? Nat Rev Genet 14:49–61 23247435 10.1038/nrg3374

[CR43] Lister DL, Thaw S, Bower MA, Jones H, Charles MP, Jones G, Smith LM, Howe CJ, Brown TA, Jones MK (2009) Latitudinal variation in a photoperiod response gene in European barley: insight into the dynamics of agricultural spread from ‘historic’specimens. J Archaeol Sci 36:1092–1098

[CR44] Lister DL, Jones H, Oliveira HR, Petrie CA, Liu X, Cockram J, Kneale CJ, Kovaleva O, Jones MK (2018) Barley heads east: genetic analyses reveal routes of spread through diverse Eurasian landscapes. PLoS One 13:e0196652 30020920 10.1371/journal.pone.0196652 PMC6051582

[CR45] Liu PP, Montgomery TA, Fahlgren N, Kasschau KD, Nonogaki H, Carrington JC (2007) Repression of AUXIN RESPONSE FACTOR10 by microRNA160 is critical for seed germination and post-germination stages. Plant J 52:133–146 17672844 10.1111/j.1365-313X.2007.03218.x

[CR46] Liu X, Zhang H, Zhao Y, Feng Z, Li Q, Yang H, Luan S, Li J, He Z (2013) Auxin controls seed dormancy through stimulation of abscisic acid signaling by inducing ARF-mediated ABI3 activation in *Arabidopsis*. Proc Natl Acad Sci U S A 110:15485–15490 23986496 10.1073/pnas.1304651110 PMC3780901

[CR47] Lönnig W-E, Saedler H (2002) Chromosome rearrangements and transposable elements. Ann Rev Genet 36:389–410 12429698 10.1146/annurev.genet.36.040202.092802

[CR48] Mallory AC, Bartel DP, Bartel B (2005) MicroRNA-directed regulation of *Arabidopsis* AUXIN RESPONSE FACTOR17 is essential for proper development and modulates expression of early auxin response genes. Plant Cell 17:1360–1375 15829600 10.1105/tpc.105.031716 PMC1091760

[CR49] Man Q, Wang Y, Gao S, Gao Z, Peng Z, Cui J (2025) Pan-genome analysis and expression verification of the maize ARF gene family. Front Plant Sci 15:1506853 40007769 10.3389/fpls.2024.1506853 PMC11850412

[CR50] Marks RA, Hotaling S, Frandsen PB, VanBuren R (2021) Representation and participation across 20 years of plant genome sequencing. Nat Plants 7:1571–1578 34845350 10.1038/s41477-021-01031-8 PMC8677620

[CR51] Marroni F, Pinosio S, Morgante M (2014) Structural variation and genome complexity: is dispensable really dispensable? Curr Opin Plant Biol 18:31–36 24548794 10.1016/j.pbi.2014.01.003

[CR52] McSteen P (2010) Auxin and monocot development. Cold Spring Harb Perspect Biol 2:a001479 20300208 10.1101/cshperspect.a001479 PMC2829952

[CR53] Team G, Mesnard T, Hardin C, Dadashi R, Bhupatiraju S, Pathak S, Sifre L, Rivière M, Kale MS, Love J (2024) Gemma: open models based on gemini research and technology. arXiv preprint arXiv:240308295

[CR54] Nguyen L-T, Schmidt HA, Von Haeseler A, Minh BQ (2015) IQ-TREE: a fast and effective stochastic algorithm for estimating maximum-likelihood phylogenies. Mol Biol Evol 32:268–274 25371430 10.1093/molbev/msu300 PMC4271533

[CR55] Okushima Y, Overvoorde PJ, Arima K, Alonso JM, Chan A, Chang C, Ecker JR, Hughes B, Lui A, Nguyen D (2005) Functional genomic analysis of the auxin response factor gene family members in *Arabidopsis thaliana*: unique and overlapping functions of ARF7 and ARF19. Plant Cell 17:444–463 15659631 10.1105/tpc.104.028316 PMC548818

[CR56] Pitz M, Baldauf JA, Piepho H-P, Yu P, Schoof H, Mason AS, Li G, Hochholdinger F (2025) Regulation of heterosis-associated gene expression complementation in maize hybrids. Genome Biol 26:291 40983969 10.1186/s13059-025-03768-3 PMC12455817

[CR57] Prigge MJ, Morffy N, de Neve A, Szutu W, Abraham-Juárez MJ, McAllister T, Jones H, Johnson K, Do N, Lavy M (2025) Comparative mutant analyses reveal a novel mechanism of ARF regulation in land plants. Nat Plants. 10.1038/s41477-025-01973-3 40216984 10.1038/s41477-025-01973-3 PMC12014491

[CR58] Purcell S, Neale B, Todd-Brown K, Thomas L, Ferreira MA, Bender D, Maller J, Sklar P, De Bakker PI, Daly MJ (2007) PLINK: a tool set for whole-genome association and population-based linkage analyses. Am J Hum Genet 81:559–575 17701901 10.1086/519795 PMC1950838

[CR59] Qiao J, Jiang H, Lin Y, Shang L, Wang M, Li D, Fu X, Geisler M, Qi Y, Gao Z (2021) A novel miR167a-OsARF6-OsAUX3 module regulates grain length and weight in rice. Mol Plant 14:1683–1698 34186219 10.1016/j.molp.2021.06.023

[CR60] Quinlan AR, Hall IM (2010) BEDTools: a flexible suite of utilities for comparing genomic features. Bioinformatics 26:841–842 20110278 10.1093/bioinformatics/btq033 PMC2832824

[CR61] Santra DK, Santra M, Allan R, Campbell K, Kidwell K (2009) Genetic and molecular characterization of vernalization genes Vrn-A1, Vrn-B1, and Vrn-D1 in spring wheat germplasm from the Pacific Northwest region of the USA. Plant Breed 128:576–584

[CR62] Schmitz RJ, Grotewold E, Stam M (2022) Cis-regulatory sequences in plants: their importance, discovery, and future challenges. Plant Cell 34:718–741 34918159 10.1093/plcell/koab281 PMC8824567

[CR63] Shabalin AA (2012) Matrix eQTL: ultra fast eQTL analysis via large matrix operations. Bioinformatics 28:1353–1358 22492648 10.1093/bioinformatics/bts163 PMC3348564

[CR64] Shen C, Wang S, Bai Y, Wu Y, Zhang S, Chen M, Guilfoyle TJ, Wu P, Qi Y (2010) Functional analysis of the structural domain of ARF proteins in rice (*Oryza sativa* L.). J Exp Bot 61:3971–3981 20693412 10.1093/jxb/erq208 PMC2935870

[CR65] Shen W, Le S, Li Y, Hu F (2016) SeqKit: a cross-platform and ultrafast toolkit for FASTA/Q file manipulation. PLoS One 11:e0163962 27706213 10.1371/journal.pone.0163962 PMC5051824

[CR66] Taketa S, Amano S, Tsujino Y, Sato T, Saisho D, Kakeda K, Nomura M, Suzuki T, Matsumoto T, Sato K (2008) Barley grain with adhering hulls is controlled by an ERF family transcription factor gene regulating a lipid biosynthesis pathway. Proc Natl Acad Sci USA 105:4062–4067 18316719 10.1073/pnas.0711034105 PMC2268812

[CR67] Tombuloglu H (2019) Genome-wide analysis of the auxin response factors (ARF) gene family in barley (*Hordeum vulgare* L.). J Plant Biochem Biotechnol 28:14–24

[CR68] Turner A, Beales J, Faure S, Dunford RP, Laurie DA (2005) The pseudo-response regulator Ppd-H1 provides adaptation to photoperiod in barley. Science 310:1031–1034 16284181 10.1126/science.1117619

[CR69] Waddington S, Cartwright P, Wall P (1983) A quantitative scale of spike initial and pistil development in barley and wheat. Ann Bot 51:119–130

[CR70] Waller F, Furuya M, Nick P (2002) OsARF1, an auxin response factor from rice, is auxin-regulated and classifies as a primary auxin responsive gene. Plant Mol Biol 50:415–425 12369618 10.1023/a:1019818110761

[CR71] Wang D, Pei K, Fu Y, Sun Z, Li S, Liu H, Tang K, Han B, Tao Y (2007) Genome-wide analysis of the auxin response factors (ARF) gene family in rice (*Oryza sativa*). Gene 394:13–24 17408882 10.1016/j.gene.2007.01.006

[CR72] Wang H, Huang Y, Li Y, Cui Y, Xiang X, Zhu Y, Wang Q, Wang X, Ma G, Xiao Q (2024) An ARF gene mutation creates flint kernel architecture in dent maize. Nat Commun 15:2565 38519520 10.1038/s41467-024-46955-9 PMC10960022

[CR73] Xing H, Pudake RN, Guo G, Xing G, Hu Z, Zhang Y, Sun Q, Ni Z (2011) Genome-wide identification and expression profiling of auxin response factor (ARF) gene family in maize. BMC Genomics 12:1–13 10.1186/1471-2164-12-178 PMC3082248 21473768

[CR74] Yu G, Wang L, Han Y, He Q (2012) ClusterProfiler: an R package for comparing biological themes among gene clusters. Omics 16:284–287 22455463 10.1089/omi.2011.0118 PMC3339379

[CR75] Zhao Z, Yin X, Li S, Peng Y, Yan X, Chen C, Hassan B, Zhou S, Pu M, Zhao J (2022) miR167d-ARF s module regulates flower opening and stigma size in rice. Rice 15:40 35876915 10.1186/s12284-022-00587-z PMC9314575

