# Supplementary figures and images for: Pan-genome analysis reveals hidden diversity and selection signatures of auxin response factors (ARFs) associated with breeding in barley
Source: Theor Appl Genet. 2026 Apr 20;139(5):127. doi: 10.1007/s00122-026-05234-5 (PMC13095972; doi:10.1007/s00122-026-05234-5)

# A

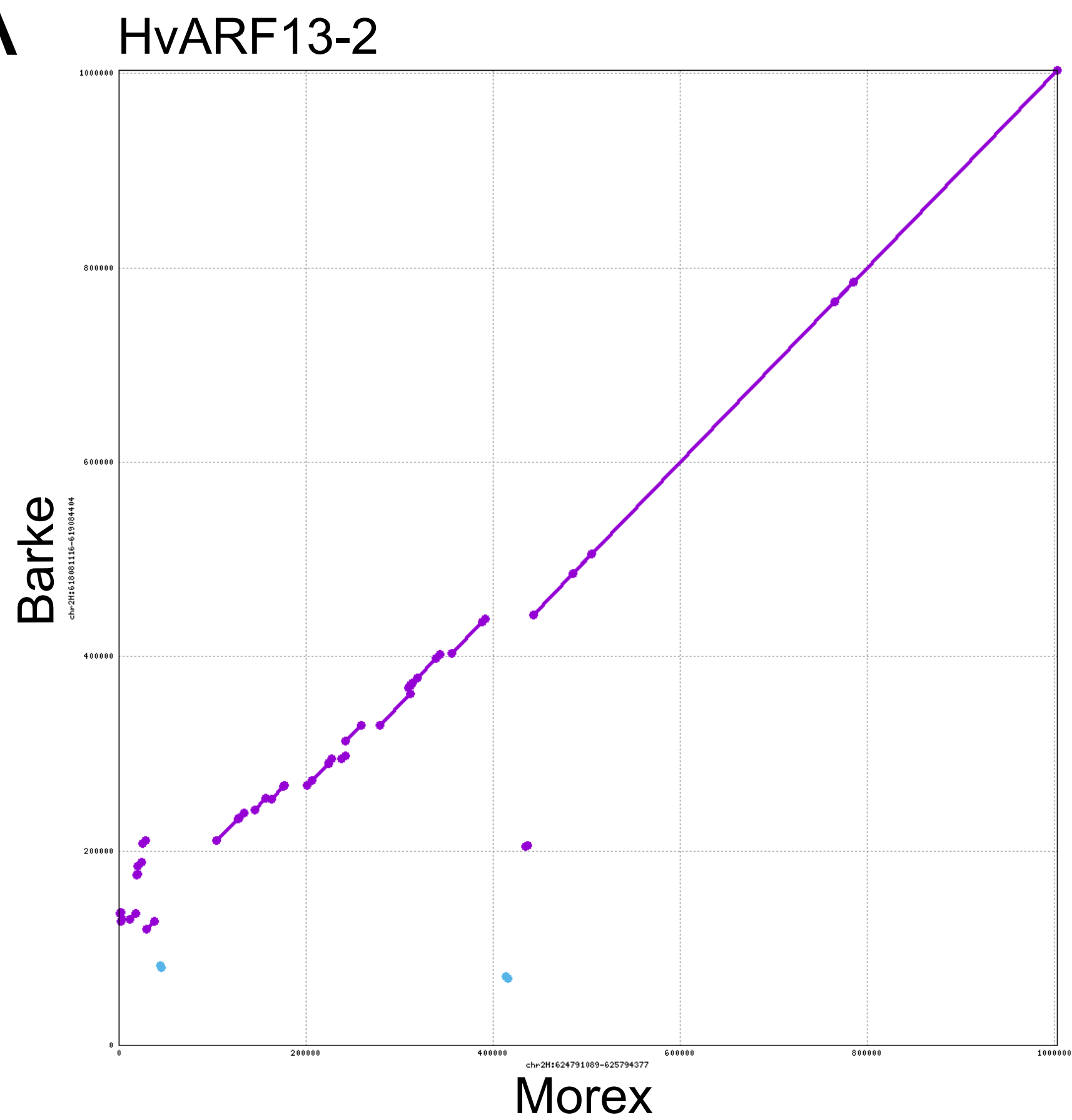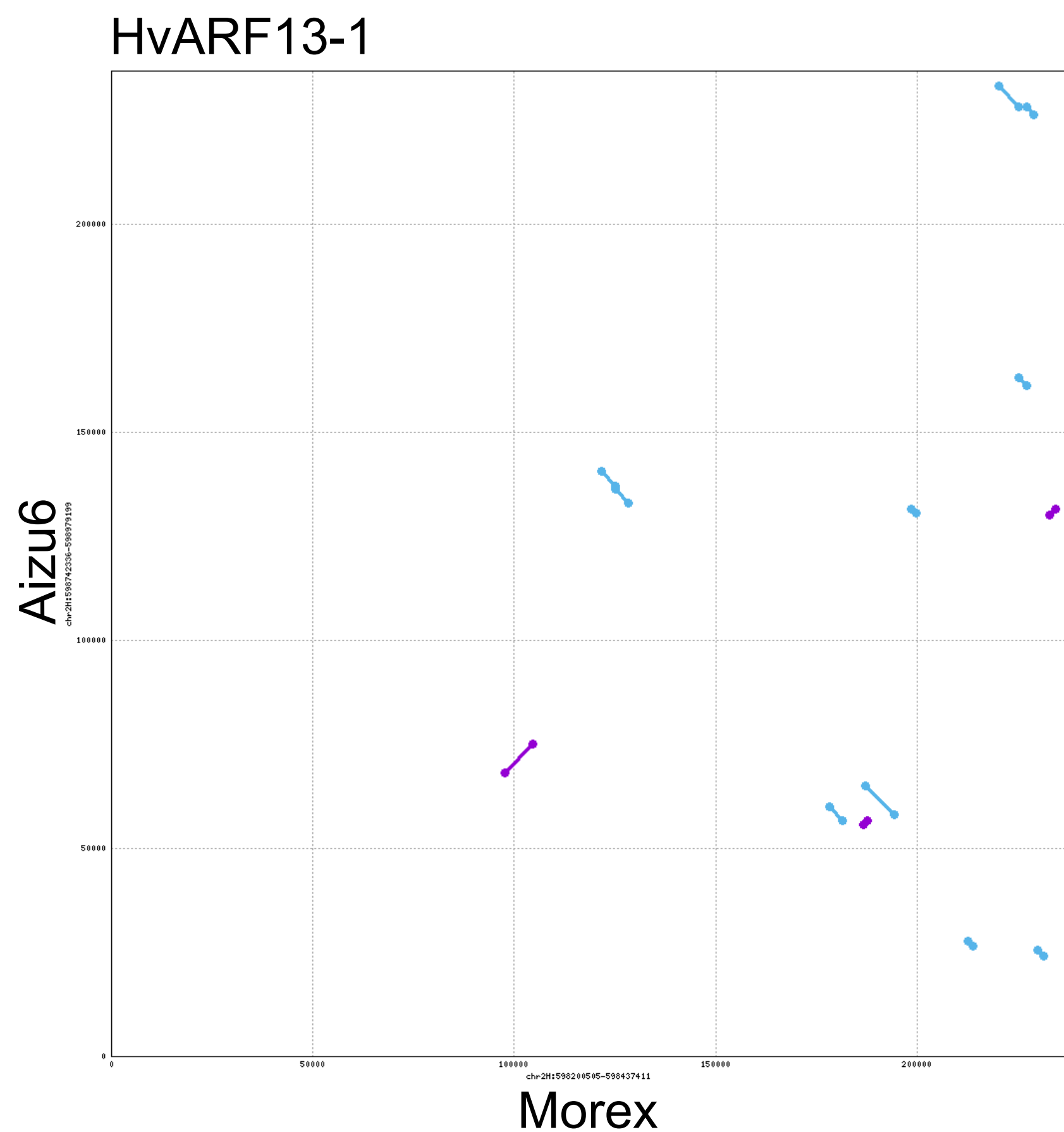

# B

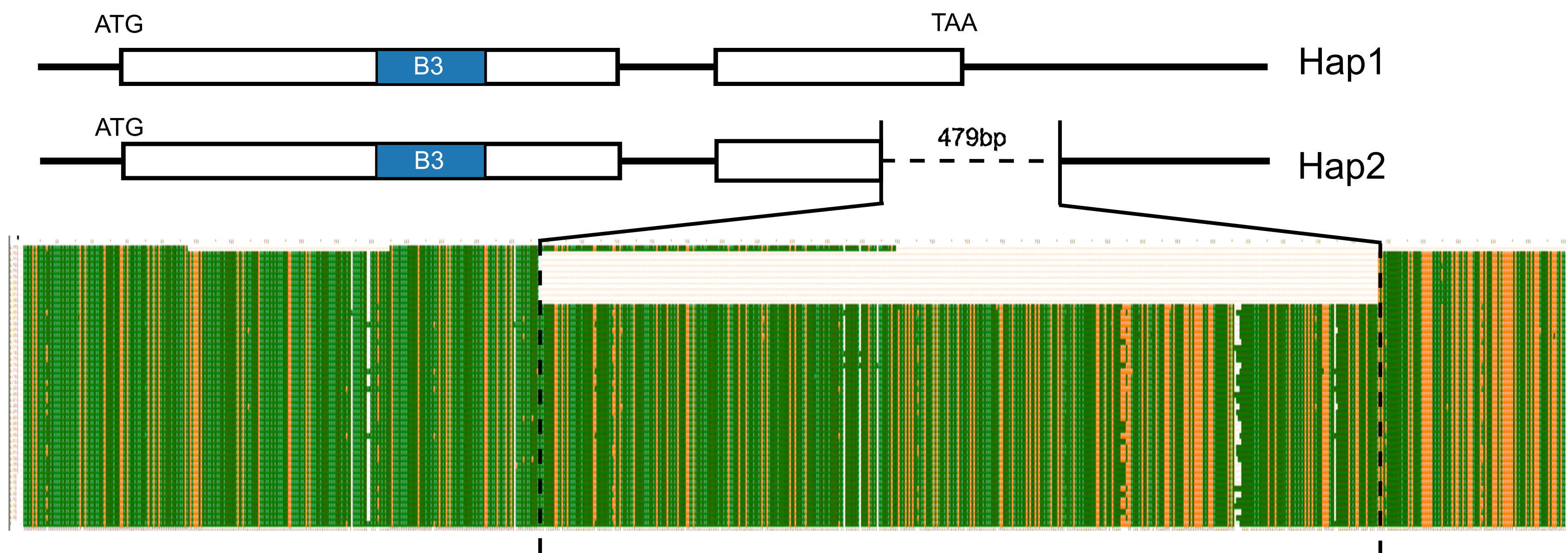

Supplement: Supplementary file 1 — Supplementary file1 (PDF 93 KB) [file 122_2026_5234_MOESM1_ESM.pdf]

A

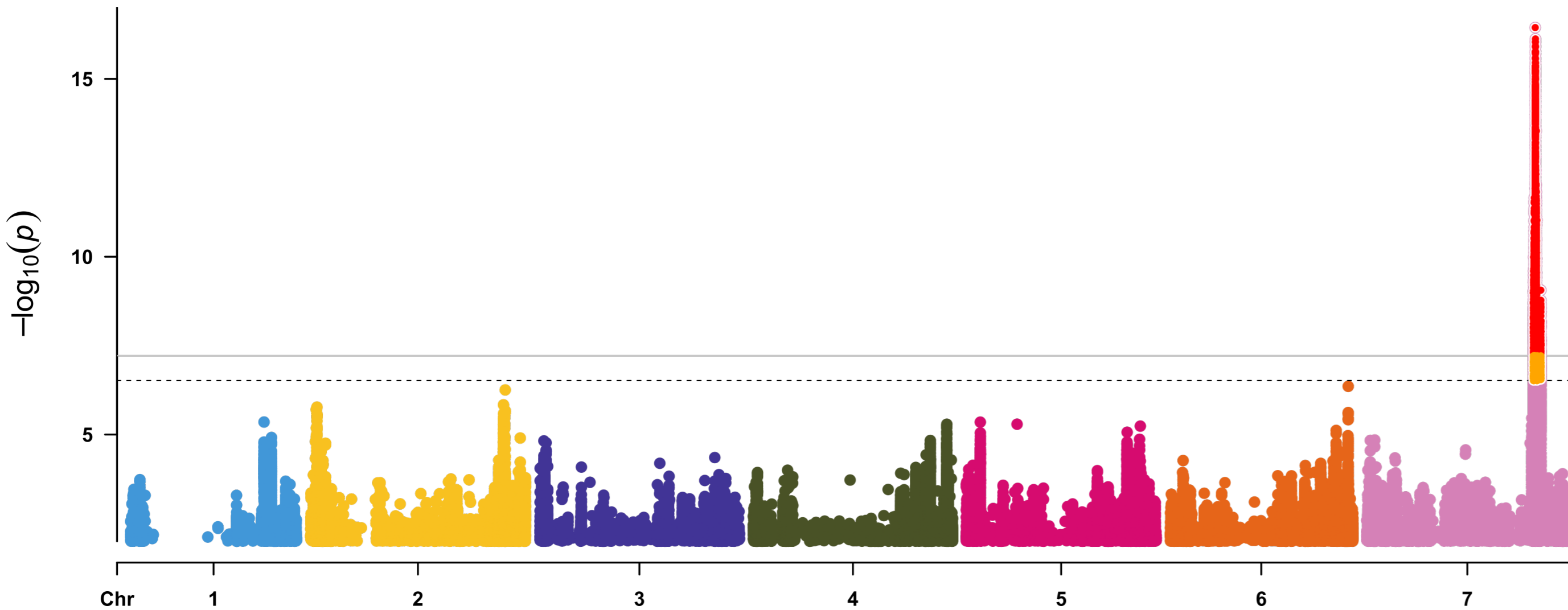

B

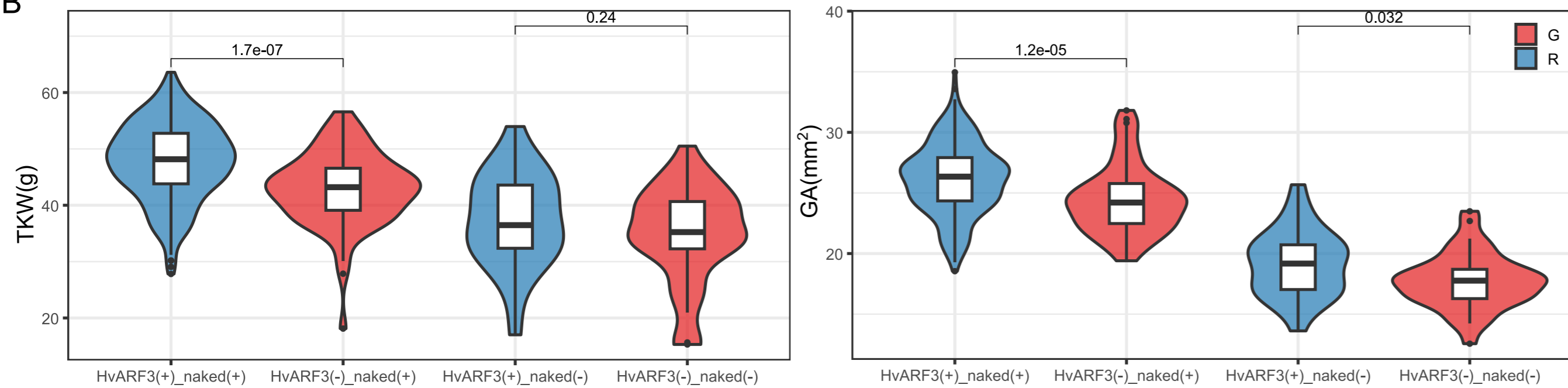

C

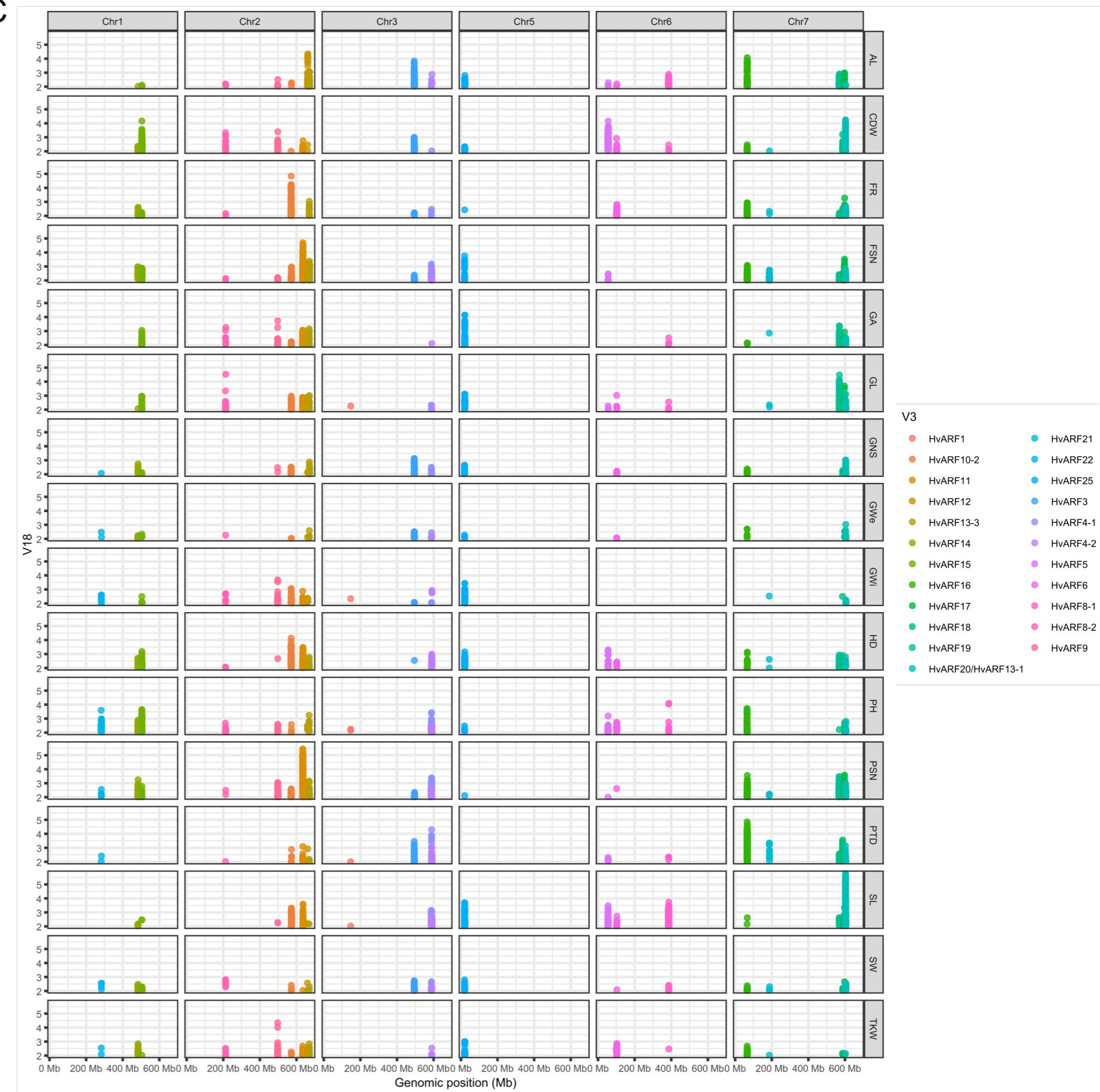

Supplement: Supplementary file 2 — Supplementary file2 (PDF 1086 KB) [file 122_2026_5234_MOESM2_ESM.pdf]

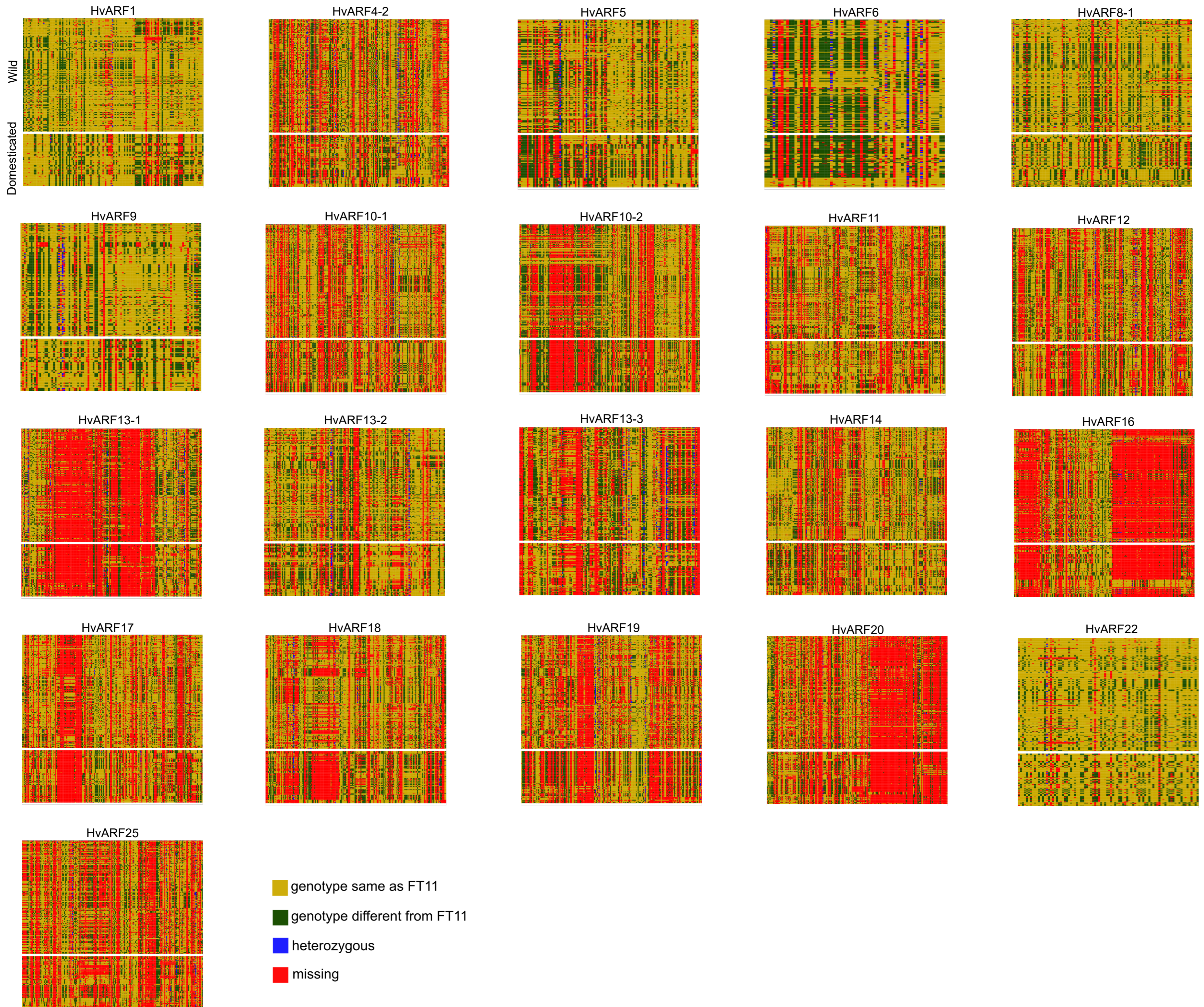

Supplement: Supplementary file 3 — Supplementary file3 (PDF 6379 KB) [file 122_2026_5234_MOESM3_ESM.pdf]

A

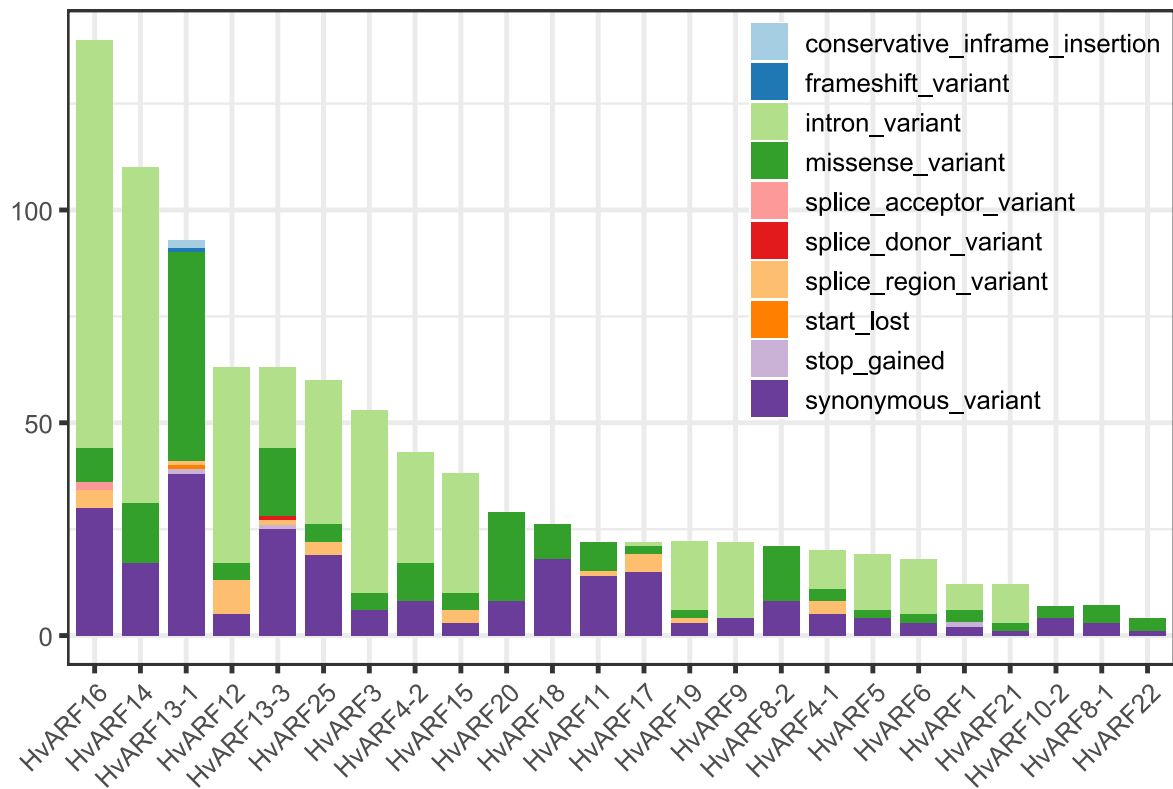

B

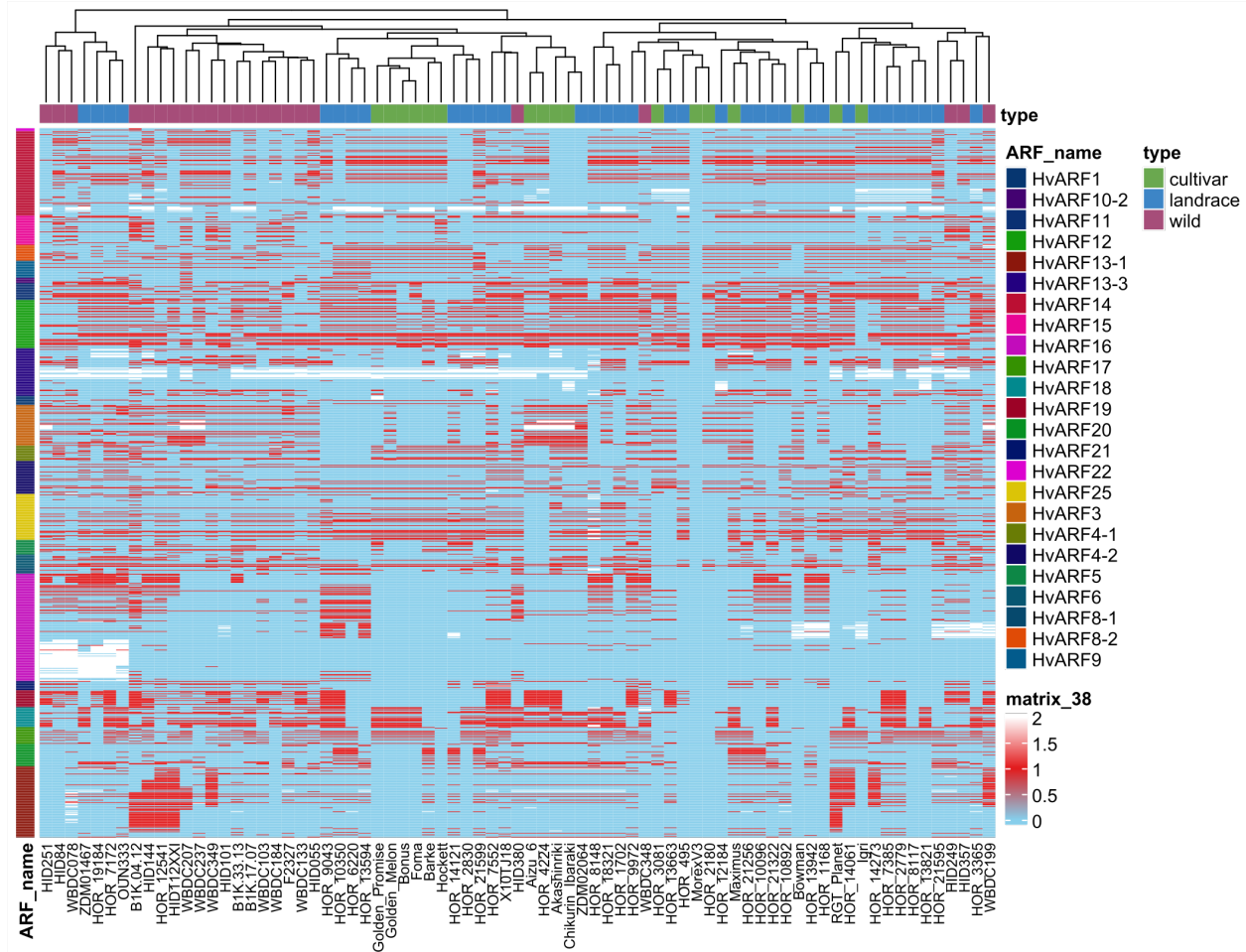

Supplement: Supplementary file 4 — Supplementary file4 (PDF 620 KB) [file 122_2026_5234_MOESM4_ESM.pdf]

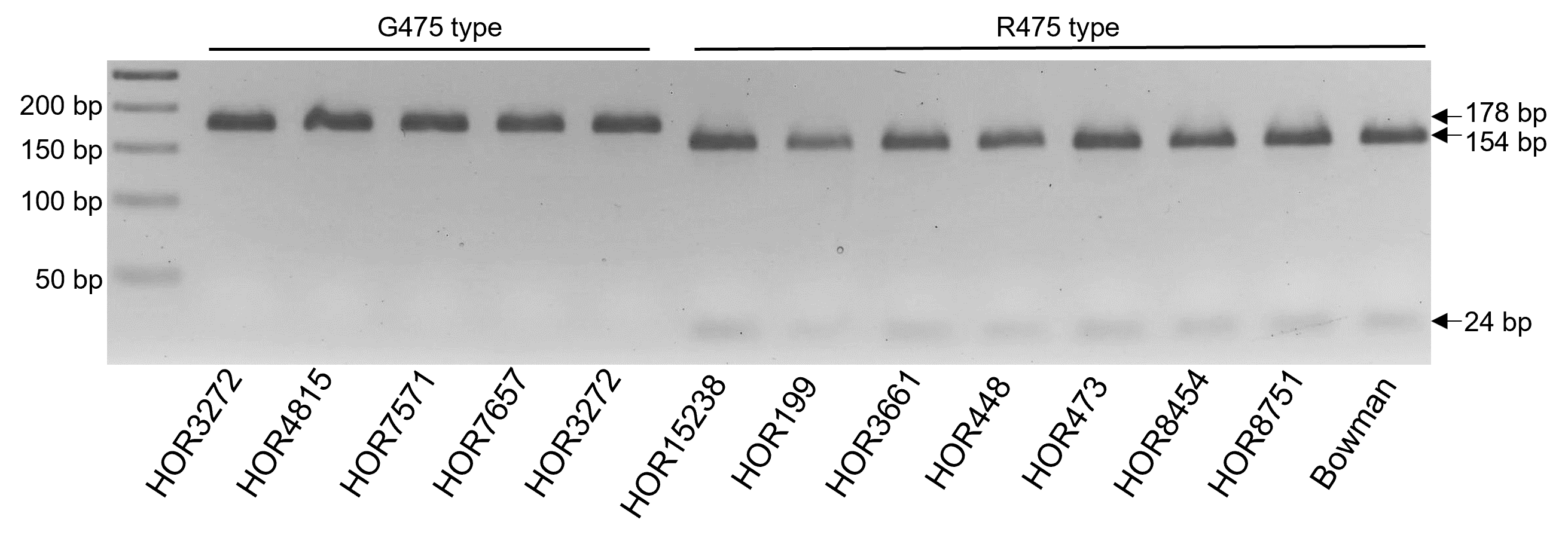

Supplement: Supplementary file 5 — Supplementary file5 (PNG 594 KB) [file 122_2026_5234_MOESM5_ESM.png]
